# Supplementary material for: Controlling of Photophysical Behavior of Rhenium(I) Complexes with 2,6-Di(thiazol-2-yl)pyridine-Based Ligands by Pendant π-Conjugated Aryl Groups
Source: Int J Mol Sci. 2022 Sep 20;23(19):11019. doi: 10.3390/ijms231911019 (PMC9569785; doi:10.3390/ijms231911019)
Supplement: Supplementary file 1 [file ijms-23-11019-s001.zip › ijms-1909043-supplementary.pdf]

CONTROLLING OF PHOTOPHYSICAL BEHAVIOUR OF RHENIUM(I) COMPLEXES WITH 2,6-DI(THIAZOL-2-YL)PYRIDINE-BASED LIGANDS BY PENDANT π-CONJUGATED ARYL GROUPS

Anna M. Maroń [1,*], Joanna Palion-Gazda [1], Agata Szłapa-Kula [1], Ewa Schab-Balcerzak [2], Mariola Siwy [2], Karolina Sulowska [3], Sebastian Maćkowski [3], and Barbara Machura[1,*]

[1] *Institute of Chemistry, University of Silesia, 9th Szkolna Street, 40006 Katowice, Poland*

[2]*Centre of Polymer and Carbon Materials, Polish Academy of Sciences, 34 M. Curie-Sklodowska Str., 41-819 Zabrze, Poland*

[3]*Nanophotonics Group, Institute of Physics, Faculty of Physics, Astronomy and Informatics, Nicolaus Copernicus University, 5 Grudziadzka Str., 87-100 Torun, Poland*

General characterization 4

Figure S1. IR spectra of 1–6 4

Table S1. CO stretching bands of 1–6 7

Figure S2. NMR spectra of 1–6 8

Crystallography 15

Table S2. Crystal data and structure refinement for 1–4 15

Table S3. Experimental and theoretical bond lengths [Å] and angles [°] for 1–4 16

Table S4. Short intramolecular contacts detected in the structure of 1–4 17

Table S5. Short π•••π interactions for 1–4 17

Table S6. Y—X•••Cg(J) (π-ring) interactions of 1–4 18

Figure S3. View of 2D supramolecular structure of 1 arising from weak π•••π and Y–C•••π type interactions (red and green dashed line). The short intramolecular contacts are indicated with the black dashed line 18

Figure S4. View of supramolecular packing of 2 arising from weak π•••π type interactions (red dashed line) and C–H•••X short contacts (black dashed line) 19

Figure S5. View of 2D supramolecular structure of 4 arising from weak π•••π and C–O•••π type interactions (red and green dashed line). The short intramolecular contacts are indicated with the black dashed line 20

DSC investigation for 2 20

Figure S6. DSC for 2 20

Electrochemistry 21

Figure S7. DPVs and CVs of 1–6 22

Table S7. Full electrochemical data for 1–6 22

Electronic absorption spectroscopy 23

Table S8. The absorption maxima and molar extinction coefficient for 1–6 in two solvents of different polarity ($CHCl_3$ and CH_3CN) and in thin film on glass substrate 23

Figure S8. UV-Vis absorption spectra of 1–6 in CH_3CN 24

Figure S9. UV-Vis absorption spectra of 1–6 in $CHCl_3$ 24

Figure S10. Absorption spectra of 1–6 vs L^1–L^6 vs hydrocarbons 25

Figure S11. The impact of the solvent polarity on the absorption properties of complexes 1–6 26

DFT and TDDFT calculations 27

Figure S12. Geometry optimization of 1–4 compared with structure determined by X-ray analysis 27

Table S9. Experimental and calculated dihedral angles [°] between the plane of polyaromatic group and central pyridine ring 27

Figure S13. The partial molecular orbital energy level diagrams for 1–6, along with plots of their frontier molecular orbitals 28

Table S10. Experimental (black line) and theoretical (blue line) absorption spectra and calculated transitions of 1 in chloroform (TD-DFT/PCM/PBE1PBE/def2-TZVPD/def2-TZVP) 29

Table S11. Experimental (black line) and theoretical (blue line) absorption spectra and calculated transitions of 2 in chloroform (TD-DFT/PCM/PBE1PBE/def2-TZVPD/def2-TZVP) 31

Table S12. Experimental (black line) and theoretical (blue line) absorption spectra and calculated transitions of 3 in chloroform (TD-DFT/PCM/PBE1PBE/def2-TZVPD/def2-TZVP)...33

Table S13. Experimental (black line) and theoretical (blue line) absorption spectra and calculated transitions of 4 in chloroform (TD-DFT/PCM/PBE1PBE/def2-TZVPD/def2-TZVP)...35

Table S14. Experimental (black line) and theoretical (blue line) absorption spectra and calculated transitions of 5 in chloroform (TD-DFT/PCM/PBE1PBE/def2-TZVPD/def2-TZVP)...37

Table S15. Experimental (black line) and theoretical (blue line) absorption spectra and calculated transitions of 6 in chloroform (TD-DFT/PCM/PBE1PBE/def2-TZVPD/def2-TZVP)...39

Stability and photostability studies ...41

Figure S14. Stability (left panels) and photostability (right panels) of 1–6 in $CHCl_3$...41

Figure S15. Stability (left panels) and photo-stability (right panel) of 1 in CH_3CN...43

Photoluminescence properties...43

Figure S16. Normalized emission spectra of 1–6 in 77K ...43

Figure S17. Decay curves of 1–6 in 77K ...44

Figure S18. Comparison of normalized emission spectra of 1–6 at 77K and at room temperature...46

Figure S19. Steady-state spectra (top) and PL decay curves with fits (bottom) of 1 in deaerated $CHCl_3$ solution at room temperature...50

Figure S20. Steady-state spectra (top) and PL decay curves with fits (bottom) of 2 in deaerated $CHCl_3$ solution at room temperature...51

Figure S21. Steady-state spectra (top) and PL decay curves with fits (bottom) of 3 in deaerated $CHCl_3$ solution at room temperature...52

Figure S22. Steady-state spectra (top) and PL decay curves with fits (bottom) of 4 in deaerated $CHCl_3$ solution at room temperature...53

Figure S23. Steady state spectra (top) and PL decay curves with fits (bottom) of 5 in deaerated $CHCl_3$ solution at room temperature...54

Figure S24. Steady-state spectra (top) and PL decay curves with fits (bottom) of 6 in deaerated $CHCl_3$ solution at room temperature 55

Table S16. The PL data for 1–6 56

Figure S25. Normalized steady-state emission spectra of 6 in aerated (black line) and deaerated (red line) $CHCl_3$ solution 57

Figure S26. PL spectra of complexes 1, 2 and 5 as thin films and in PBK:PBD matrix 57

fsTA 58

Figure S27. Fluence dependence (top) and photodamage tests (bottom) for 1, 3–4, 6 58

Table S17. Femtosecond transient absorption spectra of 1, 3–4, 6 60

Figure S28. Comparison of normalised fsTA spectra at 0.2ps of 1, 3–4 and 6 64

General characterization

Figure S1. IR spectra of **1–6**

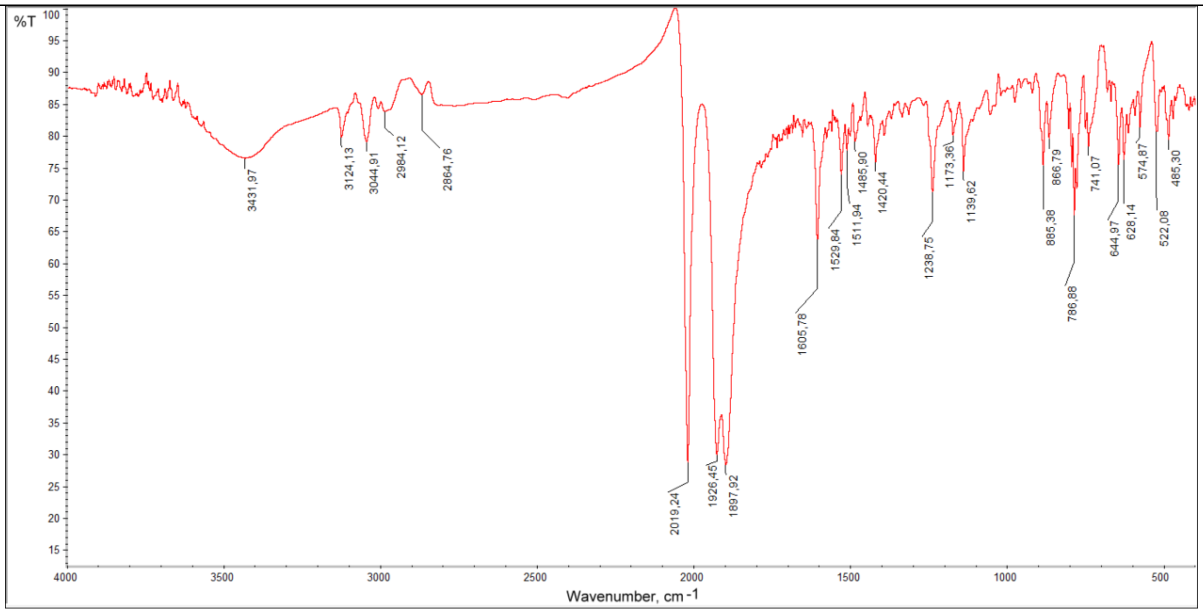

1

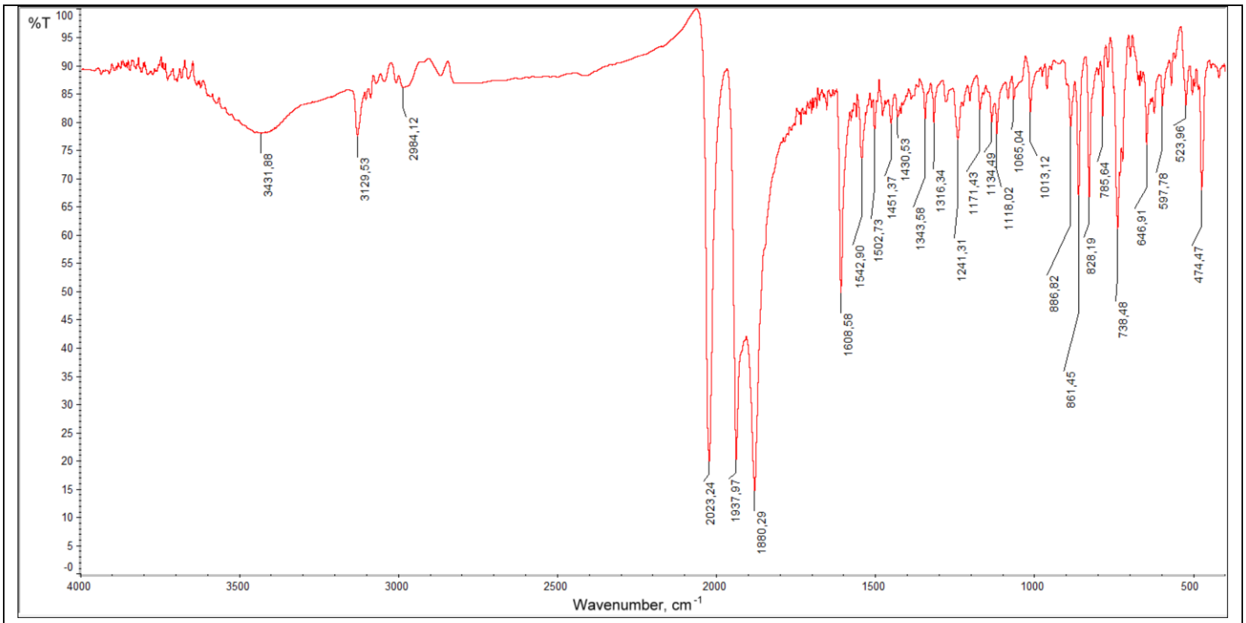
%T
Wavenumber, cm-1
3431,88
3129,53
2984,12
2023,24
1937,97
1880,29
1608,58
1542,90
1502,73
1451,37
1430,53
1343,58
1316,34
1241,31
1171,43
1134,49
1118,02
1065,04
1013,12
886,82
861,45
828,19
785,64
738,48
646,91
597,78
523,96
474,47

2

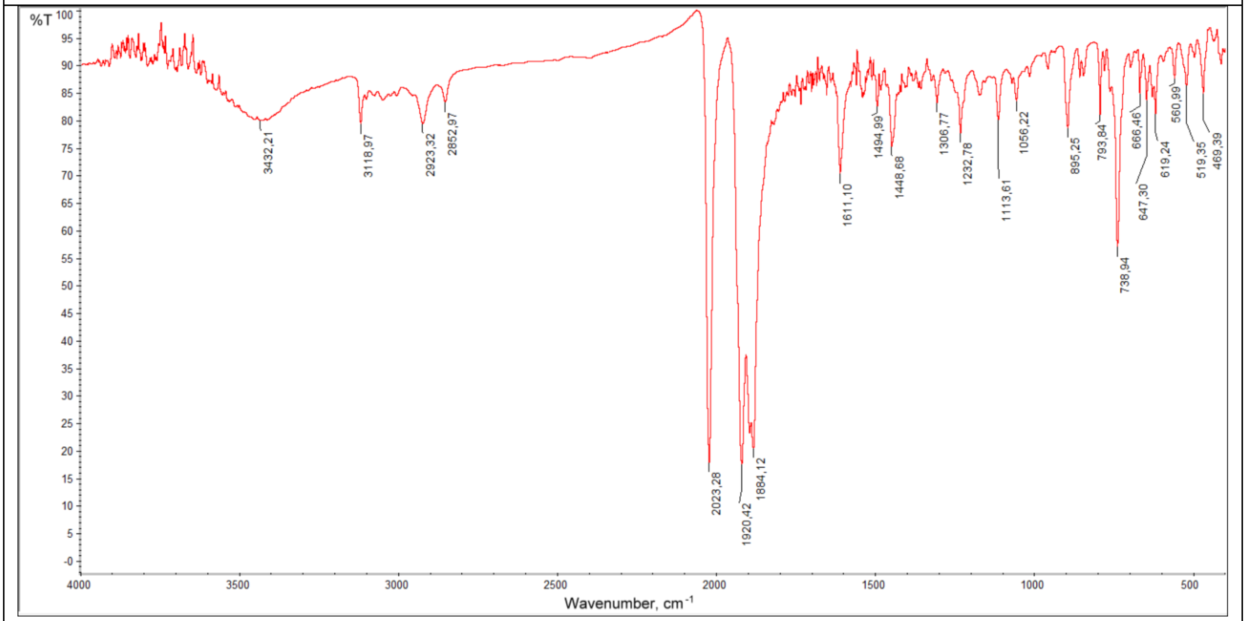
%T
Wavenumber, cm-1
3432,21
3118,97
2923,32
2852,97
2023,28
1920,42
1884,12
1611,10
1494,99
1448,68
1306,77
1232,78
1113,61
1056,22
895,25
793,84
738,94
666,46
647,30
619,24
560,99
519,35
469,39

3

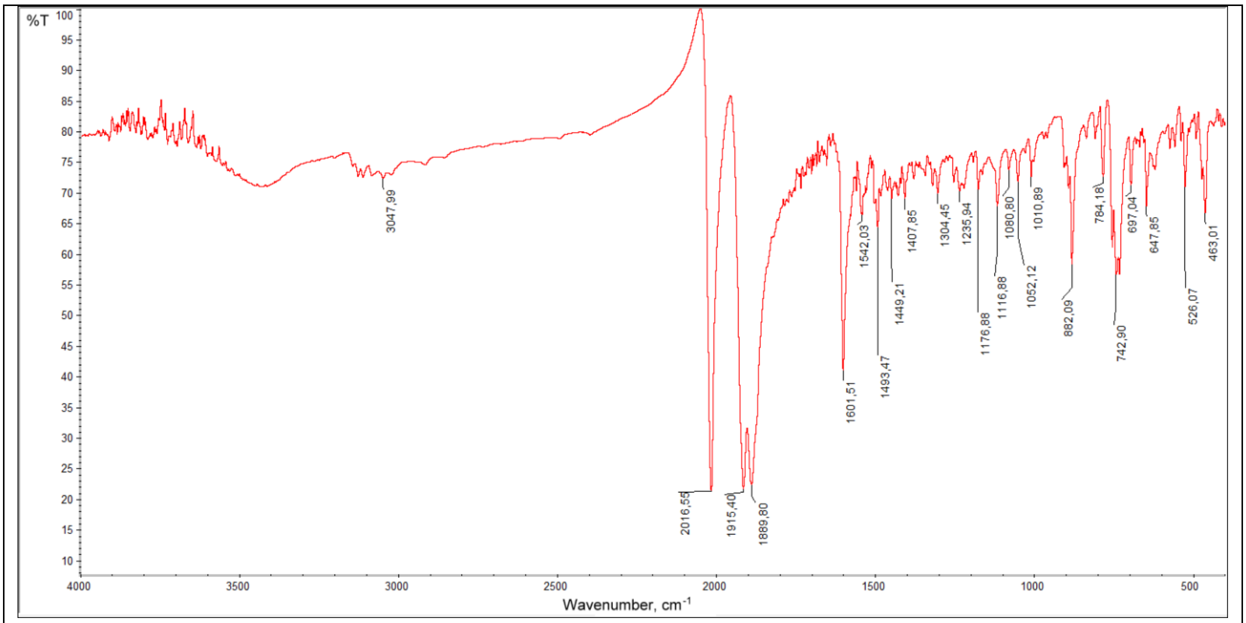
%T
100
95
90
85
80
75
70
65
60
55
50
45
40
35
30
25
20
15
10
4000
3500
3000
2500
2000
1500
1000
500
Wavenumber, cm-1
3047,99
2016,55
1915,40
1889,80
1601,51
1542,03
1493,47
1449,21
1407,85
1304,45
1235,94
1176,88
1116,88
1080,80
1052,12
1010,89
882,09
784,18
742,90
697,04
647,85
526,07
463,01

4

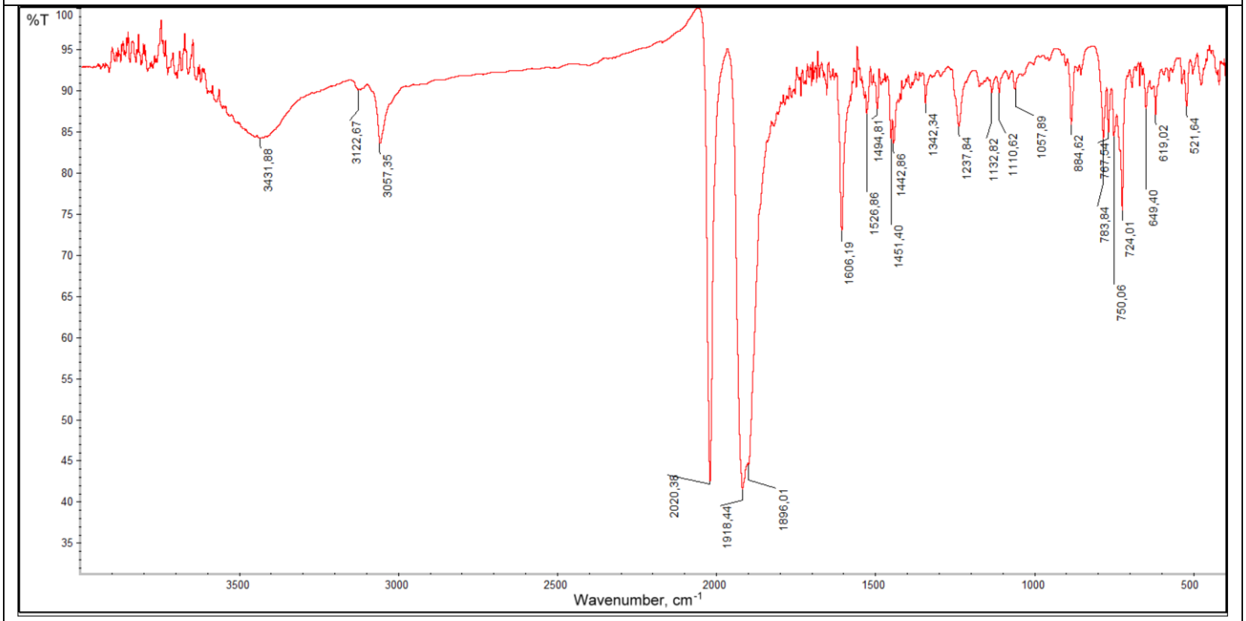
%T
100
95
90
85
80
75
70
65
60
55
50
45
40
35
3500
3000
2500
2000
1500
1000
500
Wavenumber, cm-1
3431,88
3122,67
3057,35
2020,38
1918,44
1896,01
1606,19
1526,86
1494,81
1451,40
1442,86
1342,34
1237,84
1132,82
1110,62
1057,89
884,62
783,84
767,54
750,06
724,01
649,40
619,02
521,64

5

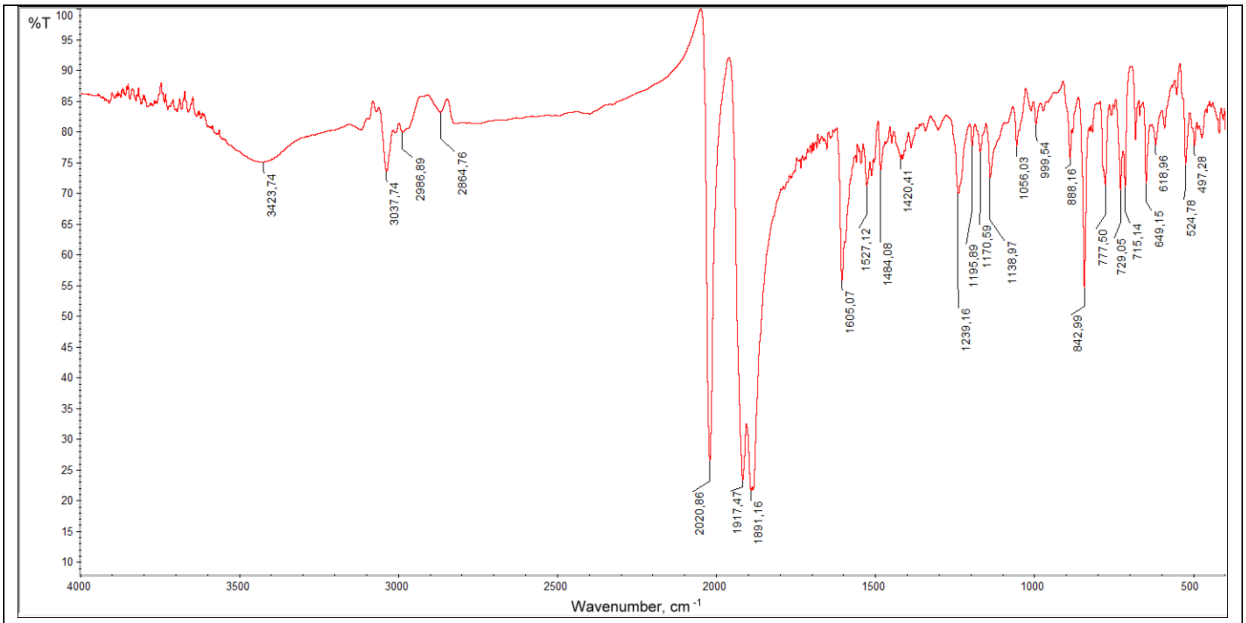

6

Table S1. CO stretching bands of **1**–**6**

Compound	ν_{CO}, cm^{-1}			
	A′(1)	A′′	A′(2)	$\nu_{CO,\ av}$
**1**	2019	1926	1898	1948
**2**	2023	1938	1880	1947
**3**	2023	1920	1884	1942
**4**	2016	1915	1890	1942
**5**	2020	1918	1896	1944
**6**	2021	1917	1891	1943

Figure S2. NMR spectra of **1**–**6**

1

^{1}H NMR (400 MHz, Acetone-d_6) δ 8.72 (s, 1H), 8.39 (d, *J* = 3.3 Hz, 1H), 8.26 (d, *J* = 3.3 Hz, 1H), 8.16 – 8.11 (m, 3H), 8.11 – 8.05 (m, 2H), 8.02 (d, *J* = 7.3 Hz, 1H), 7.80 (d, *J* = 7.1 Hz, 1H), 7.72 – 7.68 (m, 1H), 7.66 – 7.59 (m, 2H).

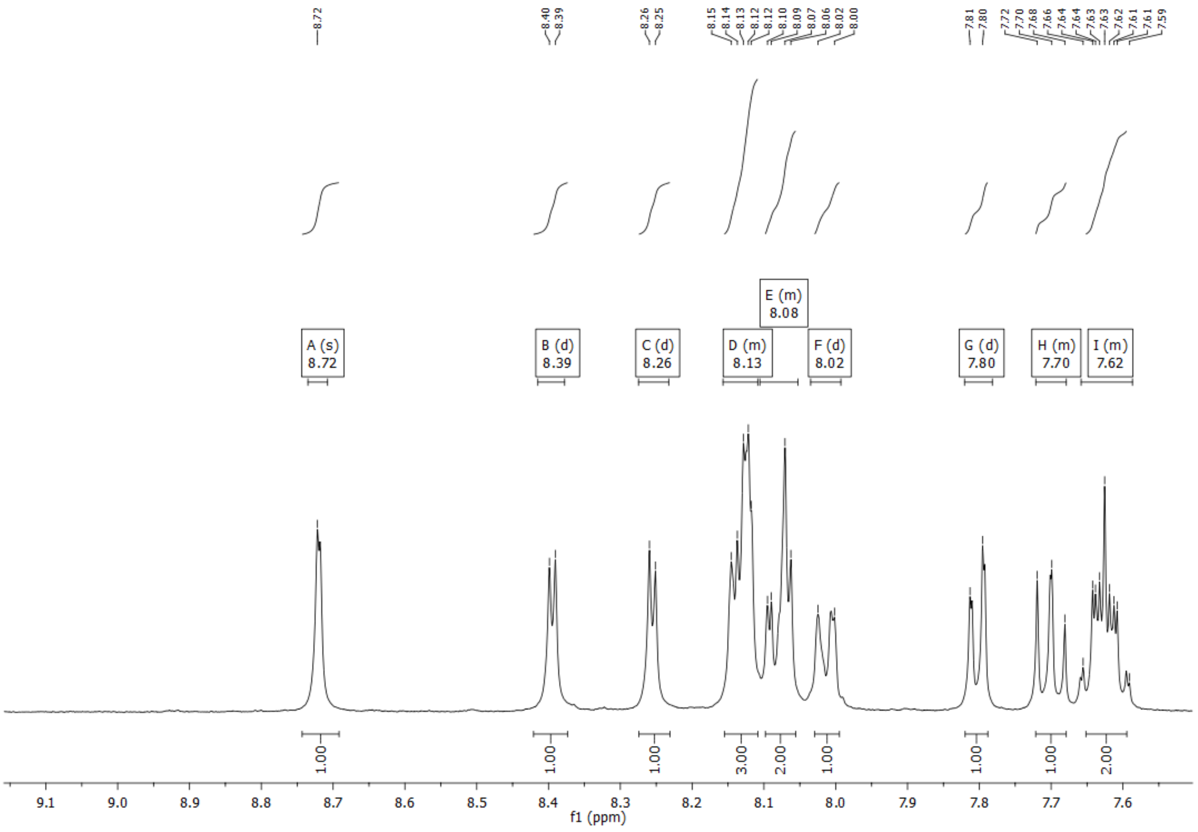

2

^{1}H NMR (400 MHz, Acetone-d_6) δ 8.95 (s, 1H), 8.75 (s, 1H), 8.39 (s, 1H), 8.37 (d, *J* = 3.3 Hz, 1H), 8.25 (d, *J* = 3.2 Hz, 1H), 8.22 – 8.19 (m, 1H), 8.17 – 8.07 (m, 4H), 8.02 (d, *J* = 7.0 Hz, 1H), 7.68 – 7.60 (m, 2H).

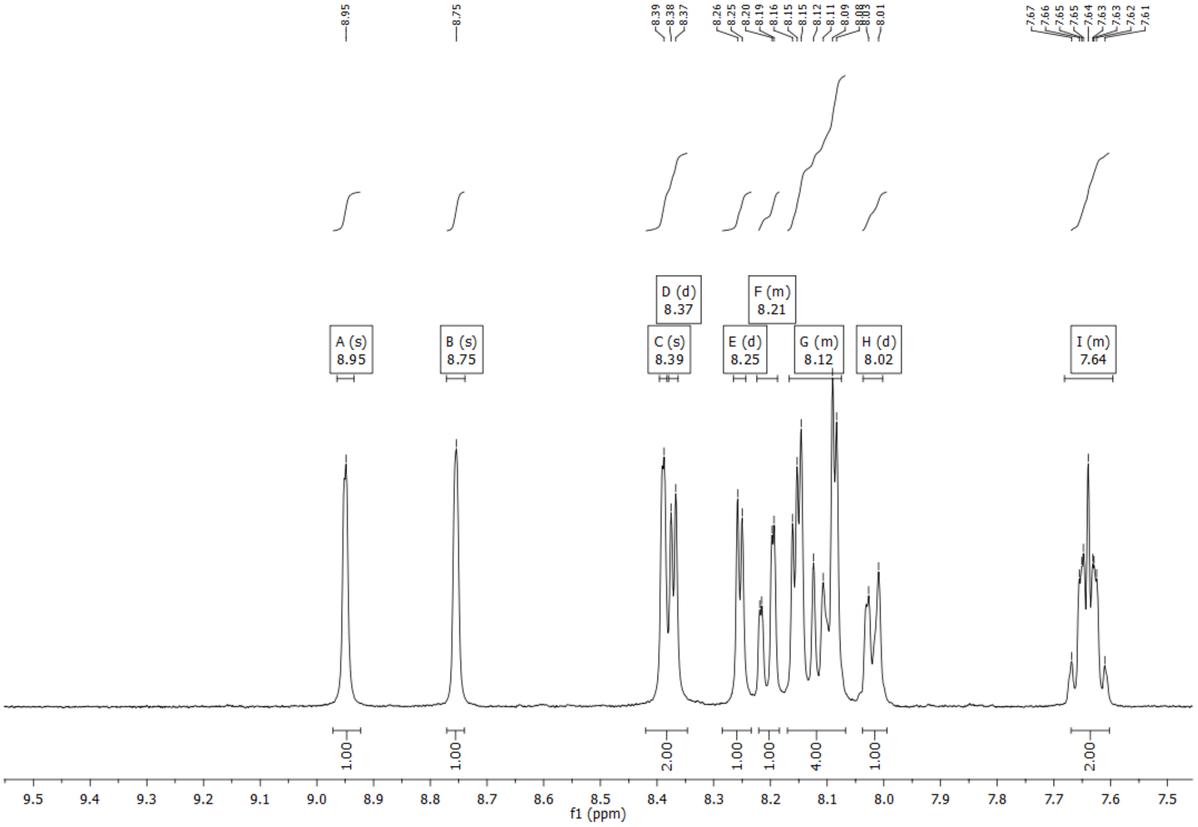

3

^{1}H NMR (400 MHz, Acetone-d_6) δ 8.79 (s, 1H), 8.73 (s, 1H), 8.43 (d, *J* = 3.2 Hz, 1H), 8.25 (d, *J* = 3.2 Hz, 1H), 8.20 (d, *J* = 8.5 Hz, 2H), 8.13 – 8.09 (m, 2H), 8.06 (d, *J* = 3.0 Hz, 1H), 7.81 (d, *J* = 8.8 Hz, 1H), 7.65 – 7.53 (m, 4H), 7.50 – 7.44 (m, 1H).

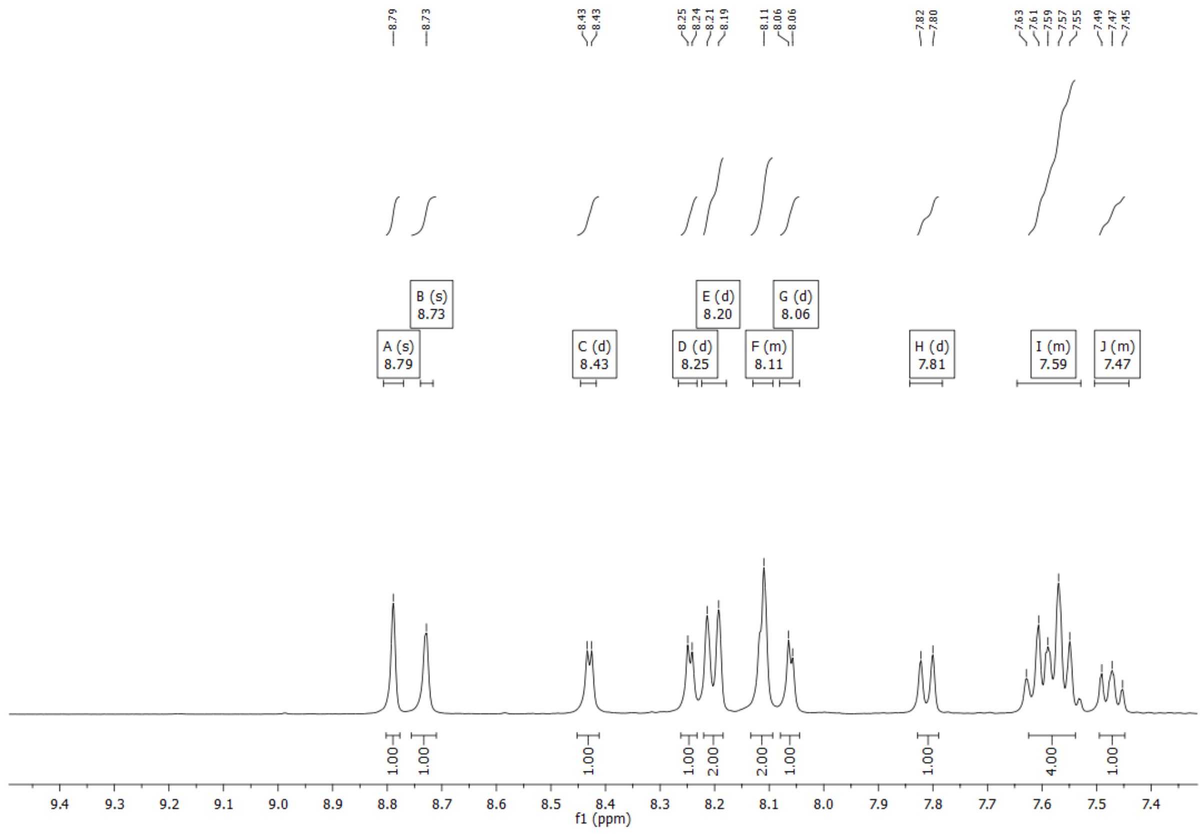

^{13}C NMR (100 MHz, Acetone-d_6) δ 197.84, 195.79, 190.80, 170.92, 165.15, 156.43, 154.28, 153.04, 145.94, 144.63, 132.15, 132.12, 131.88, 131.33, 130.05, 129.84, 129.76, 129.68, 129.03, 128.38, 128.06, 127.83, 126.59, 126.53, 126.31, 126.12, 125.78, 125.28.

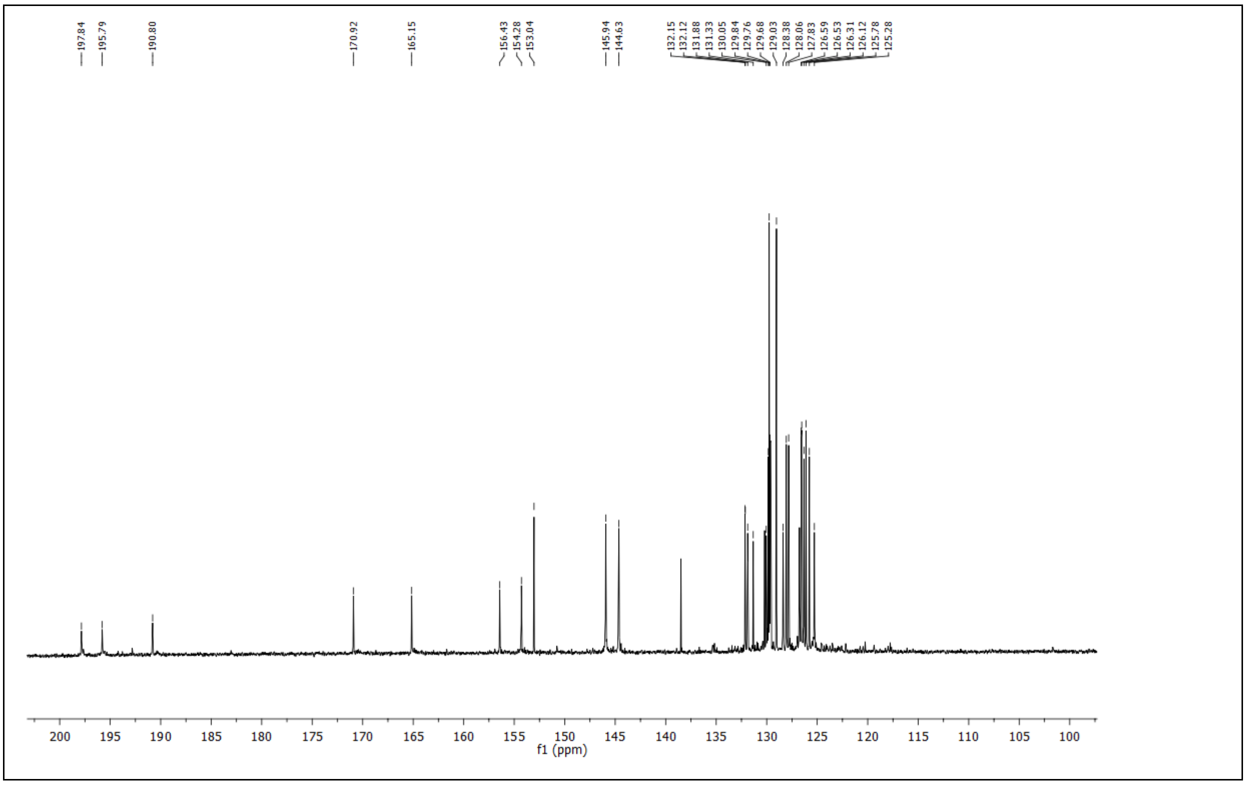

197.84
195.79
190.80
170.92
165.15
156.43
154.28
153.04
145.94
144.63
132.15
132.12
131.88
131.33
130.05
129.84
129.76
129.68
129.03
128.38
128.06
127.83
126.59
126.53
126.31
126.12
125.78
125.28
200 195 190 185 180 175 170 165 160 155 150 145 140 135 130 125 120 115 110 105 100
f1 (ppm)

**4**
^{1}H NMR (400 MHz, Acetone-d_6) δ 9.06 (s, 1H), 9.01 (s, 1H), 8.79 (s, 1H), 8.68 (s, 1H), 8.49 (s, 2H), 8.41 (d, *J* = 3.2 Hz, 2H), 8.34 (d, *J* = 9.4 Hz, 2H), 8.29 (d, *J* = 3.4 Hz, 2H), 8.23 – 8.12 (m, 10H), 7.62 – 7.59 (m, 2H).

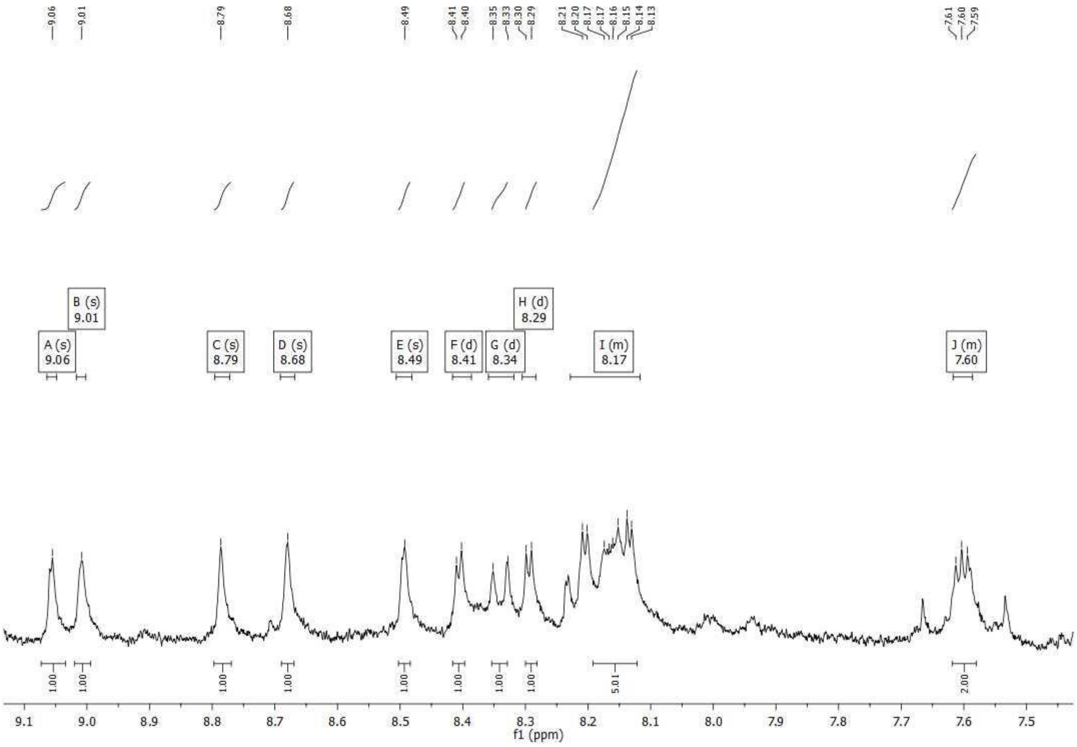

5

^{1}H NMR (400 MHz, Acetone-d_6) δ 8.99 (d, *J* = 8.2 Hz, 1H), 8.93 (d, *J* = 8.4 Hz, 1H), 8.81 (s, 1H), 8.41 (d, *J* = 3.3 Hz, 1H), 8.26 (d, *J* = 3.3 Hz, 1H), 8.21 (s, 1H), 8.17 – 8.06 (m, 4H), 8.04 (d, *J* = 8.2 Hz, 1H), 7.86 – 7.78 (m, 2H), 7.77 – 7.67 (m, 2H).

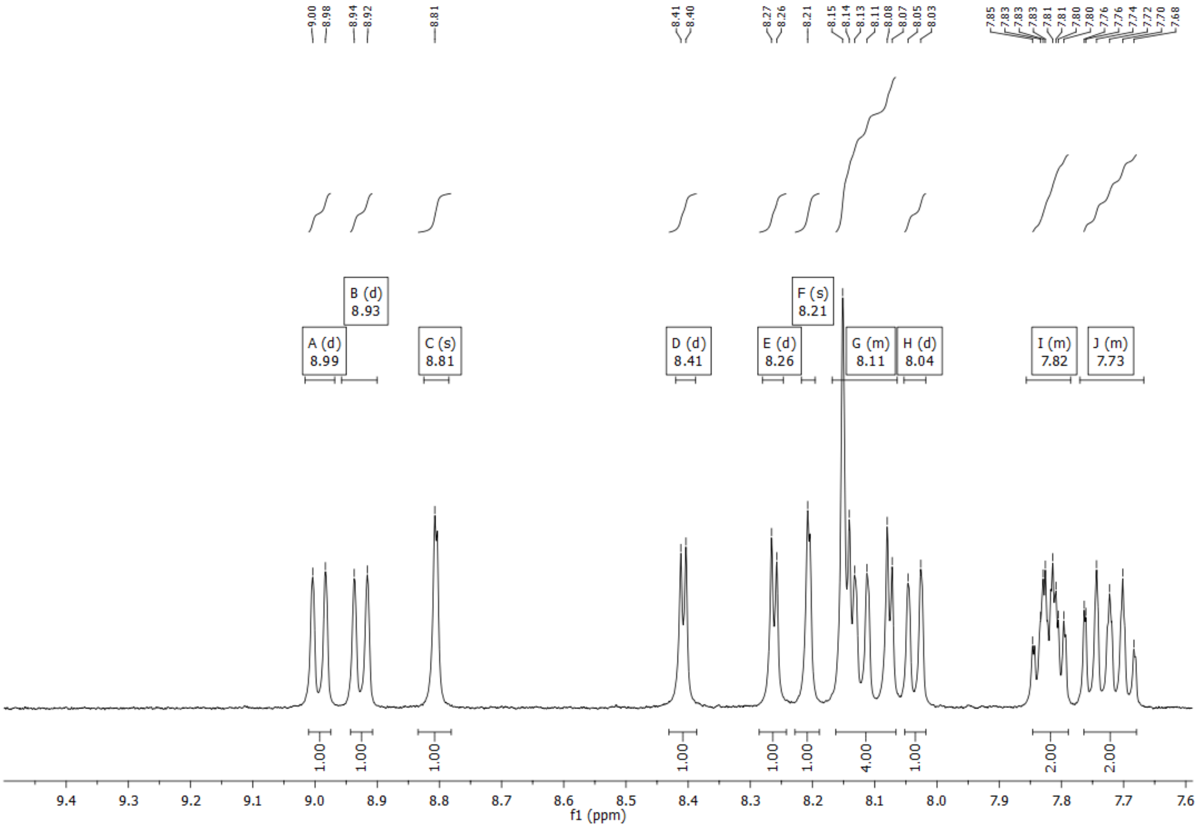

**6**
^{1}H NMR (400 MHz, Acetone-d_6) δ 8.79 (s, 1H), 8.73 (s, 1H), 8.43 (d, *J* = 3.2 Hz, 1H), 8.25 (d, *J* = 3.2 Hz, 1H), 8.20 (d, *J* = 8.5 Hz, 2H), 8.13 – 8.09 (m, 2H), 8.06 (d, *J* = 3.0 Hz, 1H), 7.81 (d, *J* = 8.8 Hz, 1H), 7.65 – 7.53 (m, 4H), 7.50 – 7.44 (m, 1H).
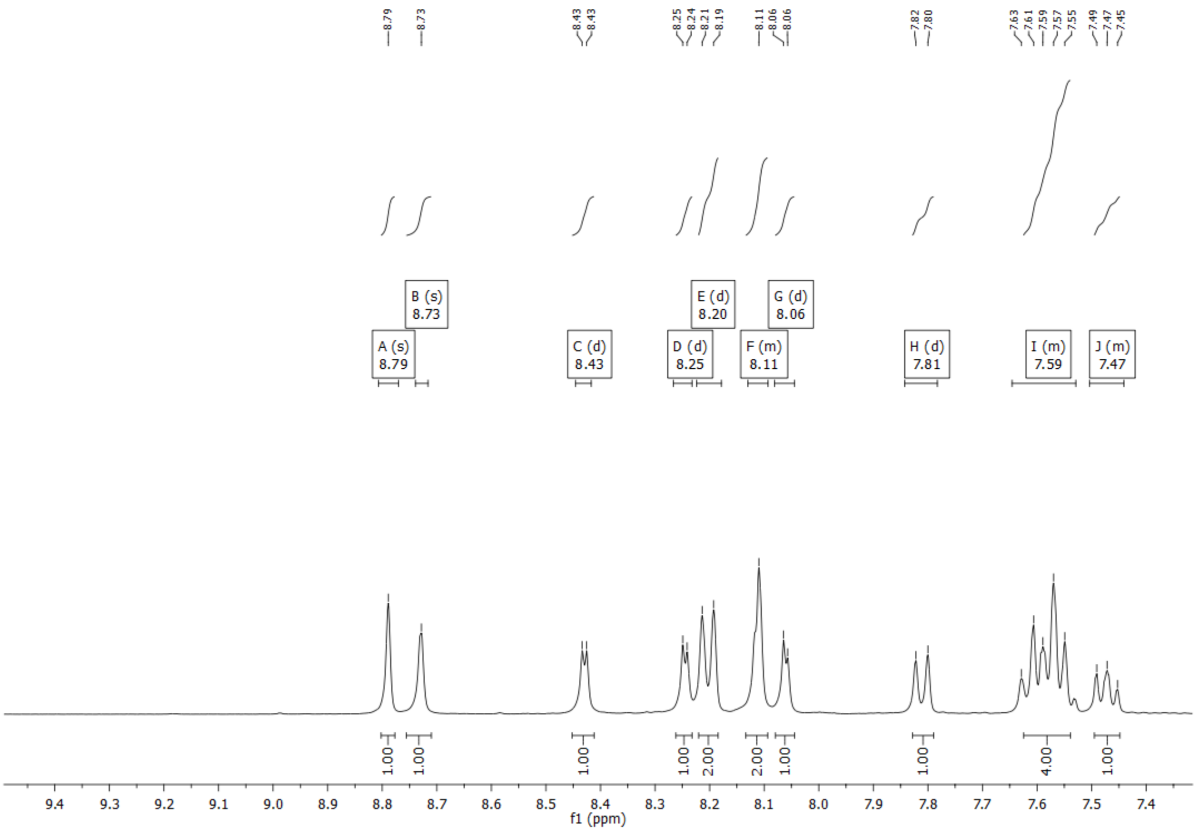

Crystallography

Table S2. Crystal data and structure refinement for **1–4**

	**1**	**2**	**3**	**4**
Empirical formula	$C_{24}H_{13}N_3O_3S_2ClRe$	$C_{24}H_{13}N_3O_3S_2ClRe$	$C_{28}H_{15}N_3O_3S_2ClRe$	$C_{35}H_{23}N_3O_3S_2ClRe$
Formula weight	677.14	677.14	727.20	819.33
Temperature [K]	295.0(2)	295.0(2)	295.0(2)	295.0(2)
Wavelength [Å]	0.71073	0.71073	0.71073	0.71073
Crystal system	*Triclinic*	*Triclinic*	*Monoclinic*	*Triclinic*
Space group	$P\bar{1}$	$P\bar{1}$	$P2_1/c$	$P\bar{1}$
Unit cell dimensions [Å, °]	a = 8.7211(3)	a = 7.7381(3)	a = 13.5194(7)	a = 11.5160(4)
	b = 12.6064(4)	b = 12.7306(6)	b = 14.6860(11)	b = 12.1388(4)
	c = 12.6861(3)	c = 15.2556(5)	c = 14.9930(8)	c = 12.7153(5)
	α = 61.221(3)	α = 65.421(4)		α = 97.774(3)
	β = 73.958(3)	β = 79.391(3)	β = 95.576(5)	β = 113.751(4)
	γ = 74.883(3)	γ = 79.071(4)		γ = 99.043(3)
Volume [Å ^{3}]	1161.26(7)	1332.52(10)	2962.7(3)	1567.46(10)
Z	2	2	4	2
Density (calculated) [g/cm^3]	1.937	1.688	1.630	1.736
Absorption coefficient [mm^{-1}]	5.560	4.845	4.365	4.136
F(000)	652.0	652.0	1408.0	804.0
Crystal size [mm]	0.17 × 0.14 × 0.10	0.22 × 0.07 × 0.05	0.21 × 0.15 × 0.1	0.20 × 0.20 × 0.13
θ range for data collection [°]	3.30 to 25.05	3.55 to 25.20	3.331 to 25.050	3.484 to 25.044
Index ranges	$-10 \le h \le 10$,	$-9 \le h \le 9$,	$-14 \le h \le 16$,	$-13 \le h \le 13$,
	$-15 \le k \le 14$,	$-12 \le k \le 15$,	$-17 \le k \le 14$,	$-13 \le k \le 14$,
	$-15 \le l \le 15$	$-18 \le l \le 18$	$-16 \le l \le 17$	$-15 \le l \le 15$
Reflections collected	11635	11574	12539	12977
Independent reflections	4109 (R_{int} = 0.0259)	4789 (R_{int} = 0.0399)	5230 (R_{int} = 0.0516)	5230 (R_{int} = 0.0516)
Completeness to 2θ=50º [%]	99.8	99.7	99.7	99.8
Max. and min. transmission	1.000 and 0.491	1.000 and 0.368	1.000 and 0.438	1.000 and 0.422
Data / restraints / parameters	4109/0/307	4789/0/307	5230/0/343	5543/0/407
Goodness-of-fit on F^2	1.050	0.973	1.101	1.062
Final R indices [I>2σ(I)]	R_1 = 0.0249	R_1 = 0.0282	R_1 = 0.0514	R_1 = 0.0354
	wR_2 = 0.0538	wR_2 = 0.0485	wR_2 = 0.1221	wR_2 = 0.0816
R indices (all data)	R_1 = 0.0292	R_1 = 0.0367	R_1 = 0.0928	R_1 = 0.0470
	wR_2 = 0.0552	wR_2 = 0.0509	wR_2 = 0.1339	wR_2 = 0.0873
Largest diff. peak and hole[eÅ^{-3}]	0.77 and -0.71	1.01 and –0.54	1.80 and –1.32	1.15 and –0.98
CCDC number	2195019	2195020	2195022	2195021

Table S3. Experimental and theoretical bond lengths [Å] and angles [°] for **1–4**

	**1**		**2**		**3**		**4**	
	**exp**	**calc**	**exp**	**calc**	**exp**	**calc**	**exp**	**calc**
**Bond lengths,** Å								
Re(1)–C(1)	1.897(5)	1.90175	1.905(5)	1.90452	1.868(12)	1.90396	1.904(7)	1.90448
Re(1)–C(2)	1.913(5)	1.92202	1.926(6)	1.92451	1.907(9)	1.92491	1.916(5)	1.92459
Re(1)–C(3)	1.908(5)	1.90664	1.893(4)	1.90475	1.877(11)	1.90494	1.976(6)	1.90475
Re(1)–N(1)	2.155(3)	2.16917	2.160(3)	2.16885	2.134(7)	2.16792	2.149(4)	2.16846
Re(1)–N(2)	2.241(3)	2.26724	2.241(3)	2.27058	2.237(6)	2.27364	2.234(4)	2.27125
Re(1)–Cl(1)	2.4897(12)	2.48912	2.4905(9)	2.48413	2.468(3)	2.48371	2.4414(16)	2.48412
C(1)–O(1)	1.152(6)	1.15189	1.155(5)	1.15057	1.173(13)	1.15051	1.151(8)	1.15057
C(2)–O(2)	1.149(5)	1.14852	1.149(6)	1.14795	1.143(10)	1.14784	1.146(6)	1.14798
C(3)–O(3)	1.141(6)	1.15325	1.157(4)	1.15478	1.160(11)	1.15470	1.029(6)	1.15475
**Bond angles, °**								
C(1)–Re(1)–C(2)	88.3(2)	86.844	87.79(19)	86.809	87.9(4)	86.729	87.5(2)	86.799
C(1)–Re(1)–C(3)	89.6(2)	89.548	95.34(15)	88.141	88.8(4)	88.186	87.9(2)	88.161
C(2)–Re(1)–C(3)	88.1(2)	88.727	89.53(18)	90.697	91.2(5)	90.666	91.7(2)	90.675
C(1)–Re(1)–N(1)	97.32(18)	96.376	95.94(16)	96.789	97.2(4)	96.690	95.6(2)	96.782
C(2)–Re(1)–N(1)	168.25(17)	174.236	173.98(15)	173.895	171.9(4)	174.051	172.93(18)	174.009
C(3)–Re(1)–N(1)	96.53(17)	95.289	95.34(15)	94.339	95.2(3)	94.299	94.77(18)	94.230
C(1)–Re(1)–N(2)	168.25(17)	169.836	169.12(15)	169.856	169.3(4)	169.707	169.95(19)	169.738
C(2)–Re(1)–N(2)	99.48(16)	102.235	101.24(15)	101.695	99.4(3)	101.881	102.22(19)	101.734
C(3)–Re(1)–N(2)	99.37(16)	95.828	98.50(13)	97.185	98.9(3)	97.249	94.35(17)	97.335
N(1)–Re(1)–N(2)	74.24(12)	74.199	74.57(12)	74.272	74.7(3)	74.261	74.46(15)	74.248
C(1)–Re(1)–Cl(1)	91.19(16)	93.830	93.78(11)	92.873	91.2(3)	93.037	94.7(2)	92.867
C(2)–Re(1)–Cl(1)	91.48(15)	91.379	89.98(13)	91.159	90.0(4)	91.106	89.54(17)	91.202
C(3)–Re(1)–Cl(1)	179.10(14)	177.324	178.59(12)	177.932	178.8(3)	177.902	177.20(14)	177.908
N(1)–Re(1)–Cl(1)	83.78(10)	83.652	85.07(8)	83.754	83.6(2)	83.869	83.87(12)	83.841
N(2)–Re(1)–Cl(1)	79.90(9)	81.522	80.30(7)	81.543	81.04(17)	81.283	82.93(10)	81.376

Table S4. Short intramolecular contacts detected in the structure of **1–4**

D—H•••A	D—H	H•••A	D•••A [Å]	D—H•••A [°]
		**1**		
C(5)–H(5)•••Cl(1)[a]	0.93	2.77	3.640(5)	155.00
C(10)–H(10)•••S(2)	0.93	2.75	3.139(5)	106.00
		**2**		
C(14)–H(14)•••O(3)[b]	0.93	2.44	3.325(7)	159.00
C(20)–H(20)•••Cl(1)[c]	0.93	2.72	3.558(5)	150.00
		**3**		
C(8)–H(8)•••S(1)	0.93	2.87	3.228(9)	105.00
C(22)–H(22)•••O(1)[c]	0.93	2.79	3.448(15)	164.00
C(25)–H(25)•••Cl(1)[d]	0.93	2.79	3.717(11)	171.00
		**4**		
C(4)–H(4)•••O(3)[e]	0.93	2.58	3.451(9)	156.00
C(8)–H(8)•••S(1)	0.93	2.85	3.226(5)	106.00
C(16)–H(16)•••Cl(1)[f]	0.93	2.73	3.644(6)	168.00

[a]Symmetry transformations used to generate equivalent atoms: (a) = 2–*x*, 1– *y*, 2–*z*; (b) = 1–*x*,2–*y*,1–*z*; (c) = 1+*x*,1/2–*y*,–1/2+*z*; (d) = *x*,1/2–*y*,1/2+*z*; (e) = 2–*x*,2-*y*,2-*z*; (f) = 2-*x*,2-*y*,1-*z*.

Table S5. Short π•••π interactions for **1–4**

Cg(I)•••Cg(J)	Cg(I)•••Cg(J) [Å]	α[°]	β[°]	γ [°]	Cg(I)-Perp [Å]	Cg(J)-Perp [Å]
			**1**			
Cg(1)•••Cg(2)[a]	3.793(3)	0	38.33	38.33	-3.644(2)	-3.644(2)
Cg(3)•••Cg(4)[b]	3.942(4)	2.8(3)	13.56	16.16	-3.786(2)	-3.832(2)
			**2**			
Cg(1)•••Cg(3)[a]	3.690(2)	8.5(2)	21.17	12.72	-3.5994(15)	-3.4413(17)
Cg(1)•••Cg(5)[a]	3.946(3)	7.8(2)	23.66	18.73	-3.737(2)	-3.6138(15)
			**4**			
Cg(2) •••Cg(6)[c]	3.734(4)	7.1(3)	22.39	15.66	3.596(2)	3.453(3)
Cg(7) •••Cg(7)[c]	3.874(3)	0	24.88	24.88	3.515(2)	3.515(2)

α = dihedral angle between *Cg*(I) and *Cg*(J); *Cg*(I)-Perp = Perpendicular distance of *Cg*(I) on ring J; *Cg*(J)-Perp = perpendicular distance of *Cg*(J) on ring I; β = angle *Cg*(I)→*Cg*(J) vector and normal to ring I; γ = angle *Cg*(I) →*Cg*(J) vector and normal to plane J;
Cg(1) = S(1)/C(5)/C(4)/N(1)/C(6);
Cg(2) = N(2)/C(7)/C(8)/C(9)/C(10)/C(11);
Cg(3) = C(15)/C(16)/C(17)/C(18)/C(19)/C(24);
Cg(4) = C(19)/C(20)/C(21)/C(22)/C(23)/C(24);
Cg(5) = C(17)/C(18)/C(24)/C(23)/C(22)/C(21);
Cg(6) = C(20)/C(21)/C(22)/C(23)/C(24)/C(25);
Cg(7) = C(15)/C(16)/C(17)/C(18)/C(27)/C(28);

Symmetry codes: (a) = 1–*x*, 1–*y*, 2–*z*; (b) = –*x*, 2–*y*, 2–*z*; (c) = 3–*x*,1–*y*, 2–*z*;

Table S6. Y—X•••Cg(J) (π-ring) interactions of **1–4**

Y(I)–X(I)•••Cg(J)	Y(I)-H(I)•••Cg(J) [Å]	X(I)-Perp [Å]	γ [°]	Y(I)–X(I)•••Cg(J) [°]
		**1**		
Re(1)–Cl(1)•••Cg(8)[a]	3.974(3)	-3.888	11.99	123.02(6)
C(2)-O(2)•••Cg(8)	3.284(5)	3.217	11.61	85.0(4)
		**2**		
C(2)-O(2)•••Cg(8)	3.409(5)	3.390	5.96	81.9(3)
		**3**		
C(2)-O(2)•••Cg(1)[b]	3.542(11)	3.186	25.89	150.3(8)
C(2)-O(2)•••Cg(8)	3.166(10)	3.162	2.90	82.4(7)
		**4**		
C(2)-O(2)•••Cg(8)	3.166(5)	-3.165	1.62	83.2(4)

γ = angle X(I)→Cg(J) vector and normal to plane J.
Cg(1) = S(1)/C(5)/C(4)/N(1)/C(6);
Cg(8) = S(2)/C(12)/N(3)/C(13)/C(14)
Symmetry codes: (a) = 1+*x*, *y*, z; (b) = 1–*x*, –1/2+*y*, 3/2–z;

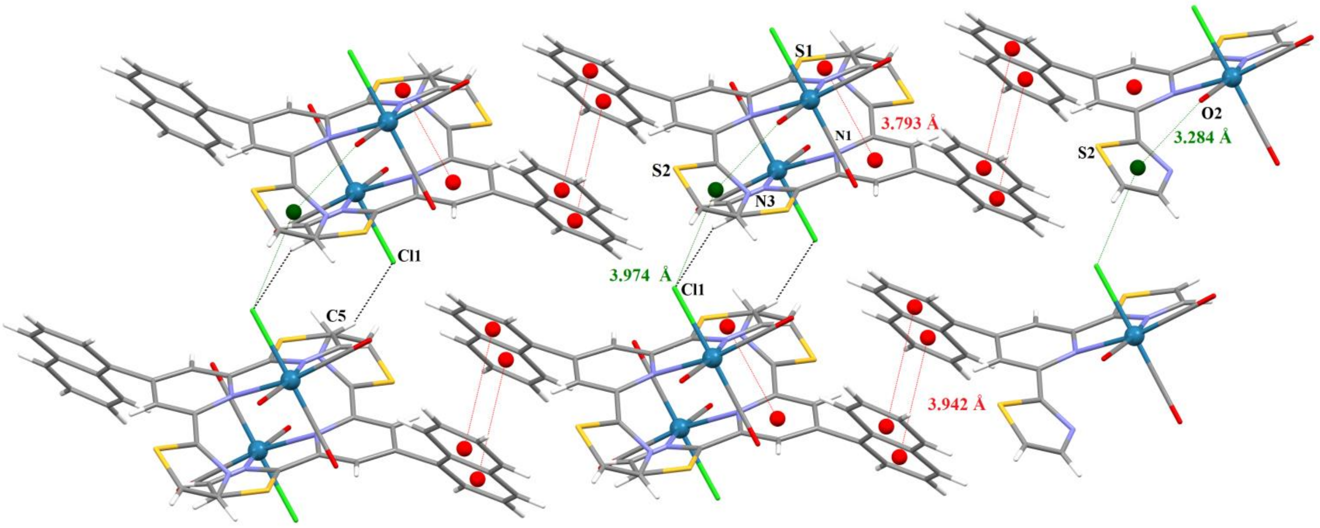

Figure S3. View of 2D supramolecular structure of **1** arising from weak π•••π and Y–C•••π type interactions (red and green dashed line). The short intramolecular contacts are indicated with the black dashed line

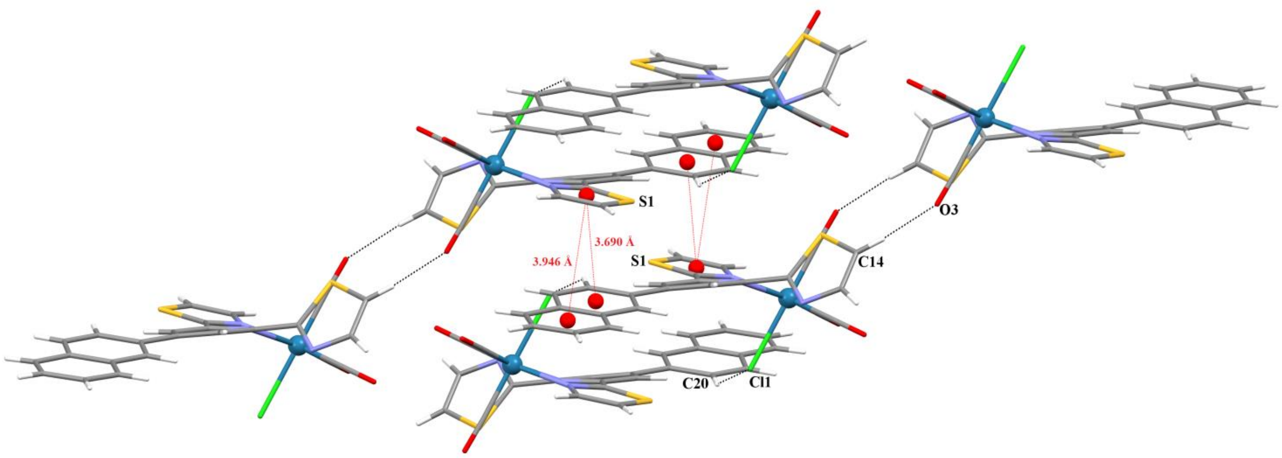

Figure S4. View of supramolecular packing of **2** arising from weak π•••π type interactions (red dashed line) and C–H•••X short contacts (black dashed line)

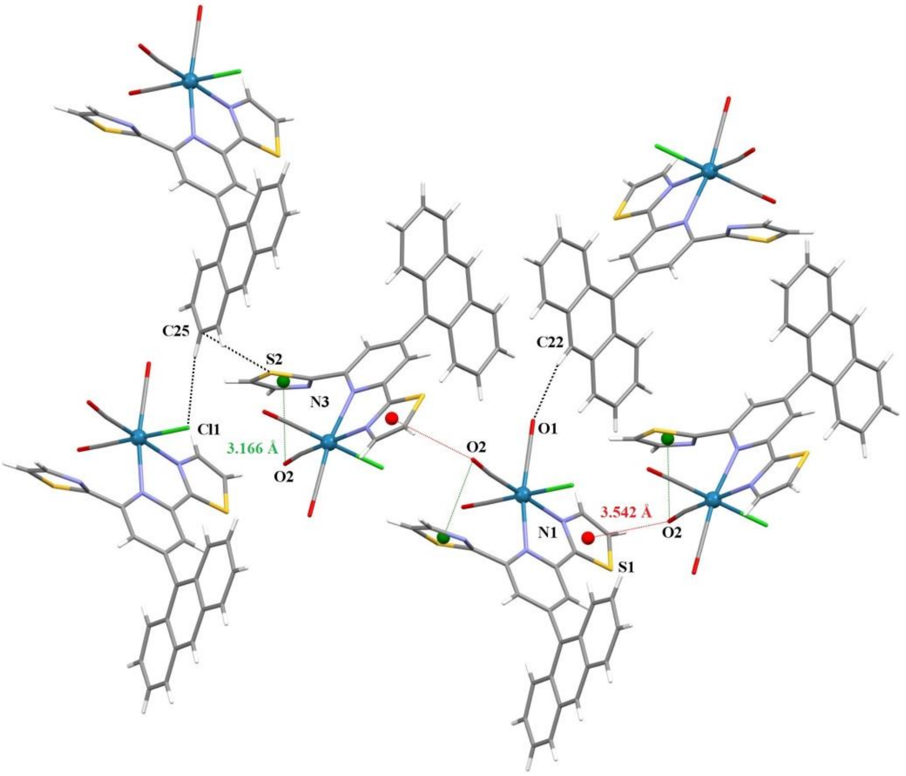

Figure S5. View of supramolecular packing of **3** arising from weak C–O•••π type interactions (red and green dashed line) and C–H•••X short contacts (black dashed line).

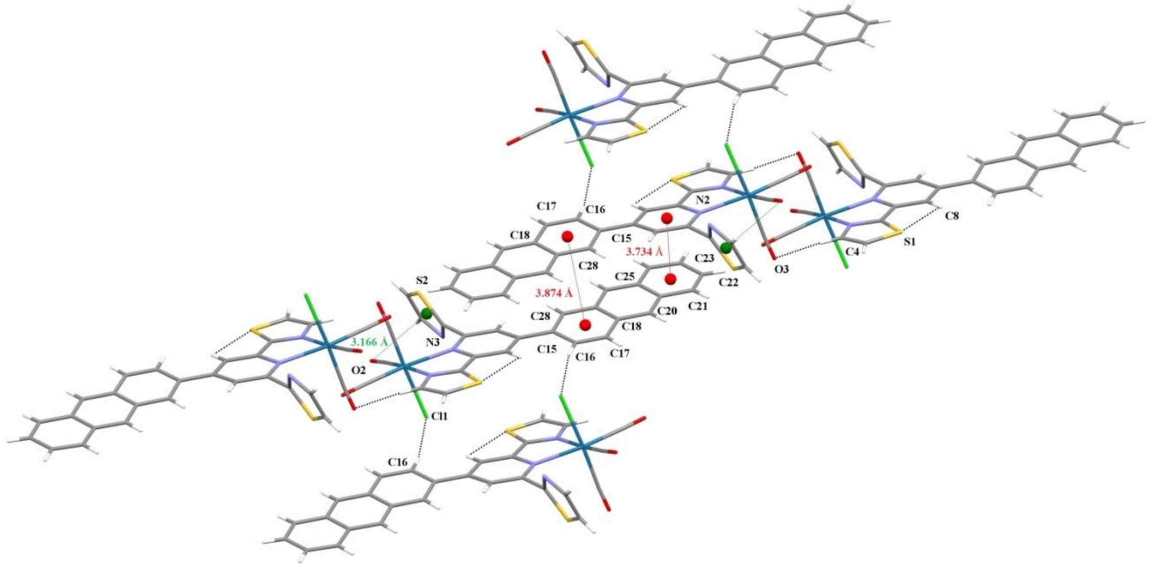

Figure S5. View of 2D supramolecular structure of **4** arising from weak π•••π and C–O•••π type interactions (red and green dashed line). The short intramolecular contacts are indicated with the black dashed line

DSC investigation for 2

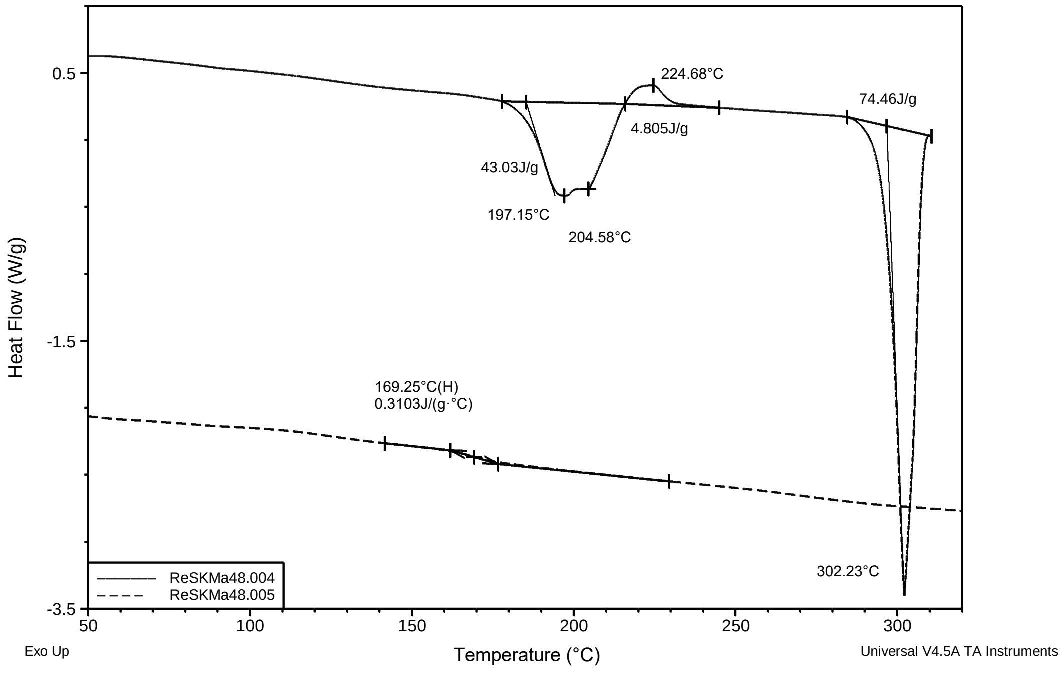

Figure S6. DSC for **2**

Electrochemistry

<table>
<tr><th>Compound</th><th>DPV</th><th>CV</th></tr>
<tr><td>1</td><td>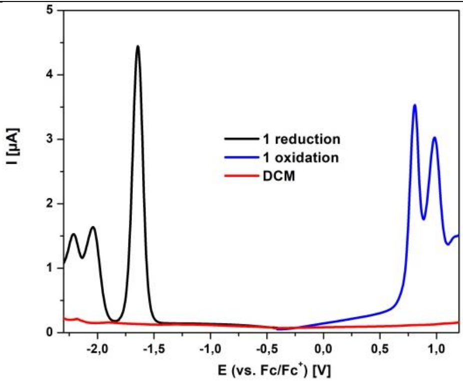
</td><td>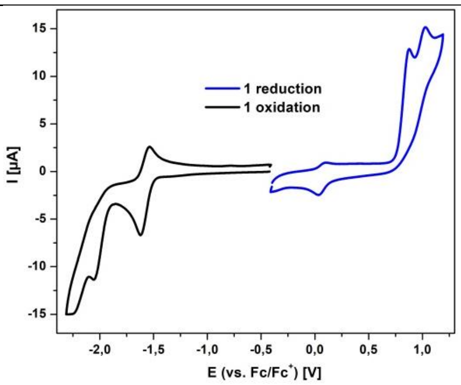
</td></tr>
<tr><td>2</td><td>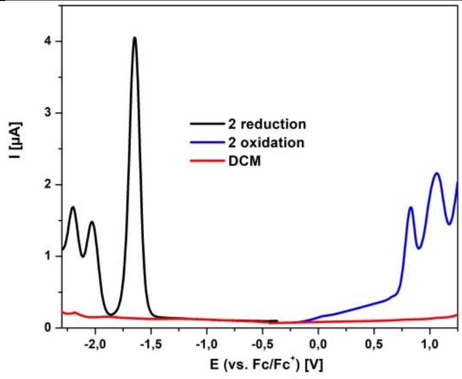
</td><td>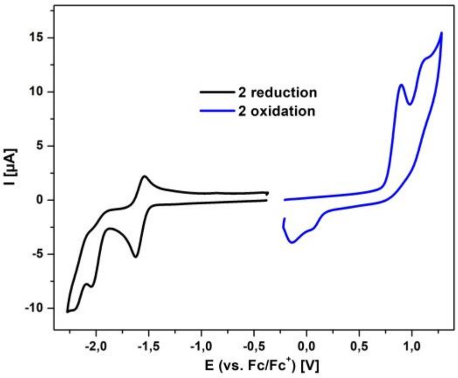
</td></tr>
<tr><td>3</td><td>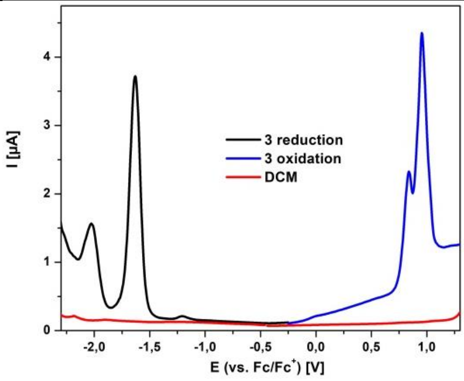
</td><td>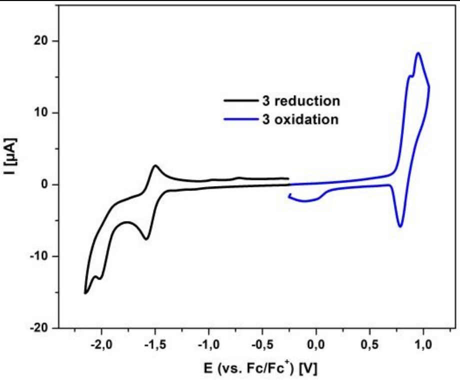
</td></tr>
<tr><td>4</td><td>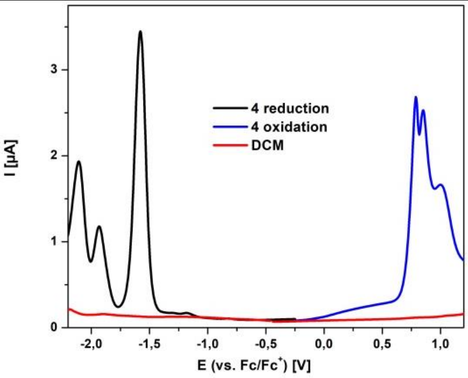
</td><td>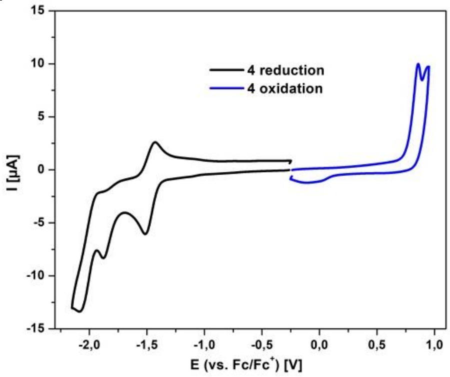
</td></tr>
</table>

**5**	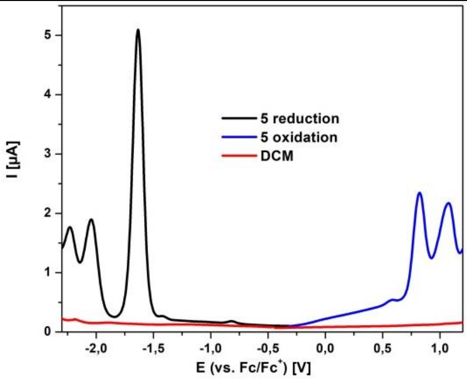	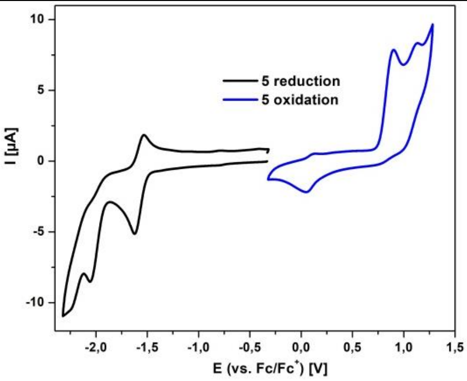
**6**	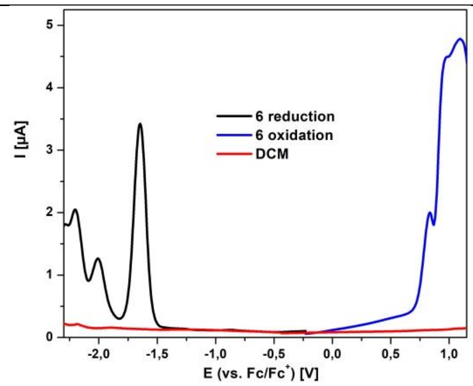	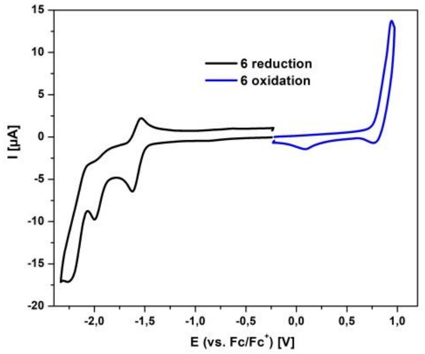

Figure S7. DPVs and CVs of **1**–**6**

Table S7. Full electrochemical data for **1–6**

Compound	E^{onset}_{1red} [V]	E^{onset}_{2red} [V]	E^{onset}_{3red} [V]	E^{onset}_{1ox} [V]	E^{onset}_{2ox} [V]	IP[a] (CV)	EA[b] (CV)	$E_{g(CV)}$[c] [eV]
1	-1.50 (-1.53)[d]	-1.91 (-1.92)[d]	-2.11 (-2.14)[d]	0.74 (0.71)[d]	0.94 (0.91)[d]	-5.84	-3.60	2.24
2	-1.51 (-1.54)[d]	-1.91 (-1.92)[d]	-2.11 (-2.13)[d]	0.74 (0.73)[d]	0.98 (0.94)[d]	-5.84	-3.59	2.25
3	-1.49 (-1.53)[d]	-1.87 (-1.90)[d]	—	0.74 (0.75)[d]	0.90 (0.94)[d]	-5.84	-3.61	2.23
4	-1.48 (-1.47)[d]	-1.80 (-1.82)[d]	-1.96 (-2.02)[d]	0.73 (0.70)[d]	0.88 (0.83)[d]	-5.83	-3.62	2.21
5	-1.52 (-1.53)[d]	-1.93 (-1.93)[d]	-2.13 (-2.16)[d]	0.75 (0.72)[d]	1.00 (0.95)[d]	-5.85	-3.58	2.27
6	-1.52 (-1.54)[d]	-1.87 (-1.90)[d]	-2.09 (-2.11)[d]	0.76 (0.73)[d]	—	-5.86	-3.58	2.28

[a] IP = -5.1 - E_{ox}; [b] EA = -5.1 – E_{red}; [c] $Eg_{(CV)} = E_{ox\ (onset)} - E_{red\ (onset)}$; [d]determined by DPV measurement

Electronic absorption spectroscopy

Table S8. The absorption maxima and molar extinction coefficient for **1**–**6** in two solvents of different polarity ($CHCl_3$ and CH_3CN) and in thin film on glass substrate

	**$CHCl_3$**	**CH_3CN**	**Thin film**
		**λ [nm] (ε · 10^3 [M·cm^{-1}])**	
**1**	415 (4.10), 326 (16.30), 258 (17.61)	390 (8.49), 324 (27.91), 253 (30.02)	400, 329
**2**	416 (5.53), 324 (24.93), 276 (25.91), 267 (sh, 25.43)	389 (7.06), 318 (26.44), 273 (28.90)	400, 323
**3**	430 (5.04), 386 (7.85), 365 (8.07), 333 (14.43),	416 (5.05), 384 (8.53), 364 (9.34), 329 (14.88), 252 (101.97)	431, 388,334,
**4**	438 (14.61), 373 (sh 12.53), 355 (sh 22.47), 326 (53.08), 300 (37.59)	424 (20.18), 322 (74.68), 294 (64.06), 251 (111.24)	436, 332,
**5**	413 (9.33), 323 (33.09), 297 (28.67), 254 (96.49)	393 (10.39), 325 (32.92), 295 (32.72), 252 (103.80)	400, 326,
**6**	426 (12.08), 378 (sh 9.53), 331 (25.94), 272 (sh 23.78), 264 (26.24)	407 (15.05), 334 (33.32), 270 (sh 32.42), 260 (34.39)	452, 338, 323,

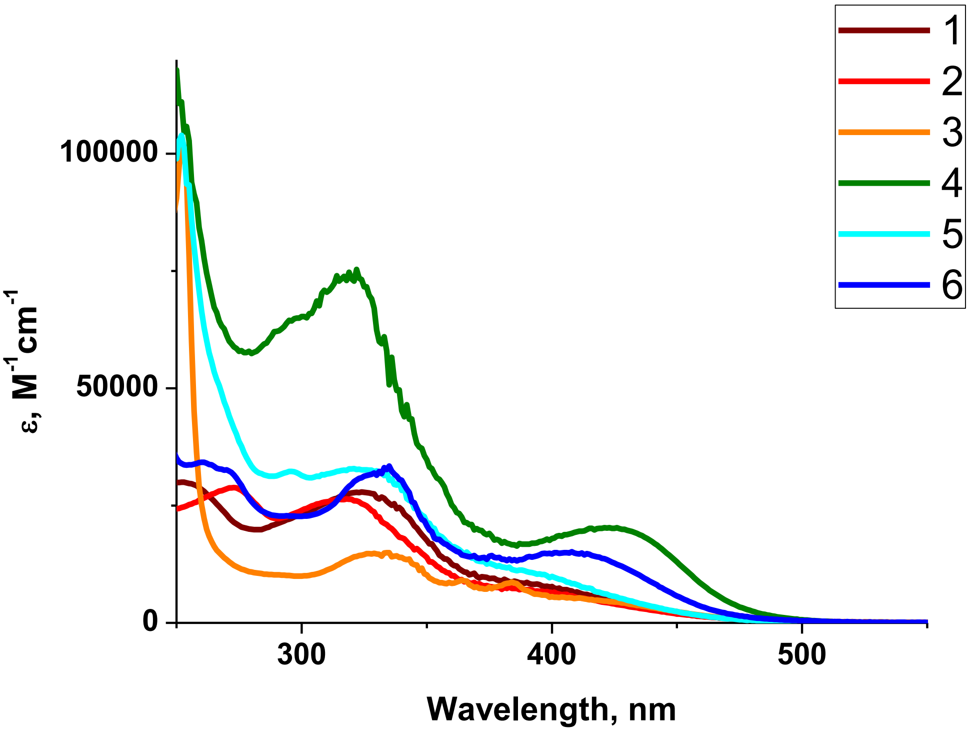

Figure S8. UV-Vis absorption spectra of **1–6** in CH_3CN

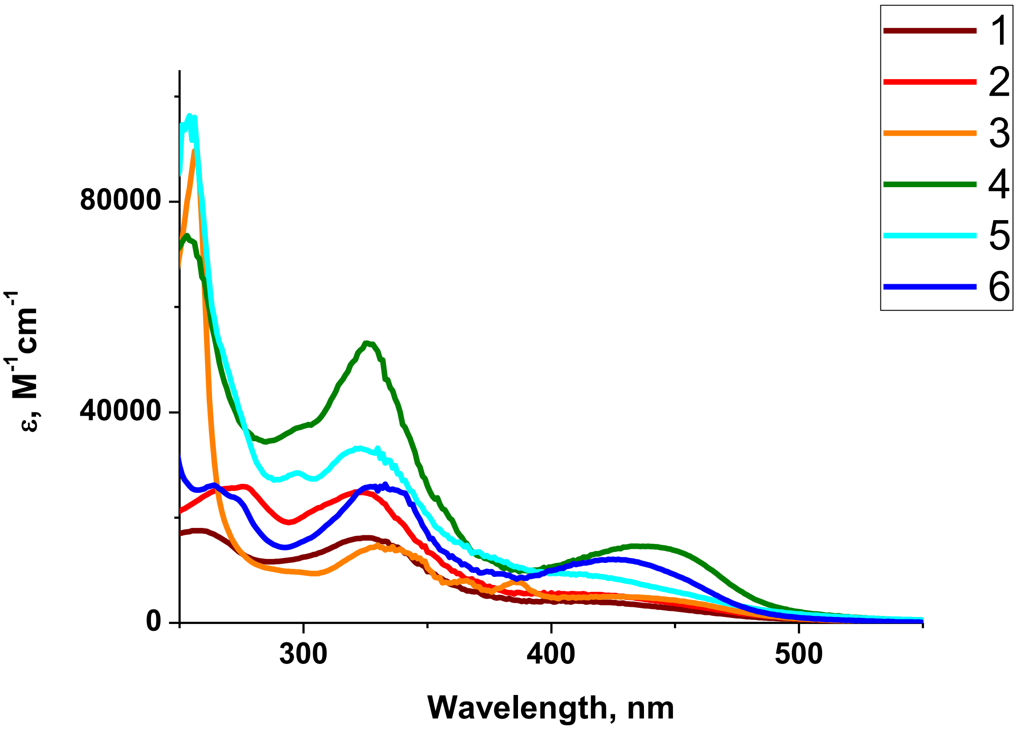

Figure S9. UV-Vis absorption spectra of **1–6** in $CHCl_3$

Figure S10. Absorption spectra of **1–6** vs **L**^{1}–**L**^{6} vs hydrocarbons

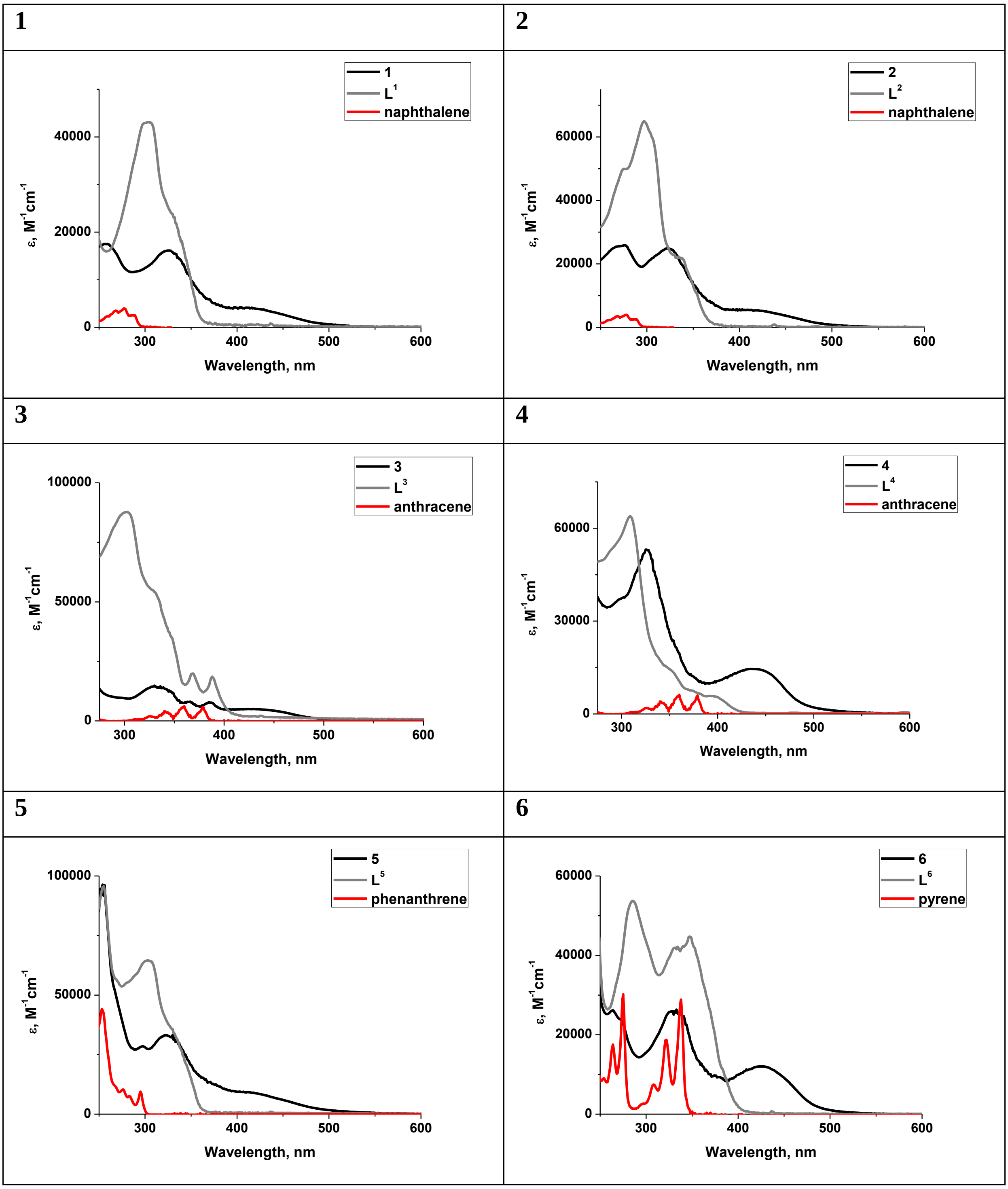

Figure S11. The impact of the solvent polarity on the absorption properties of complexes **1**–**6**

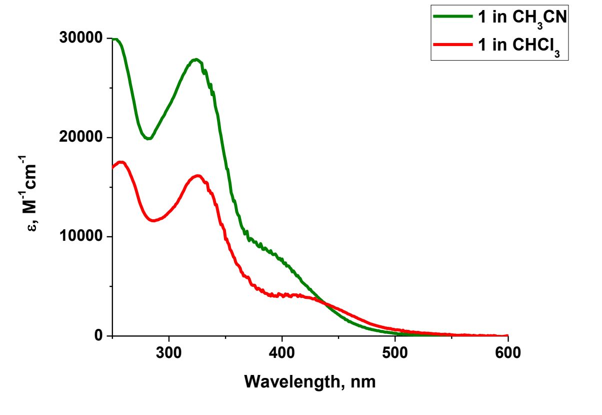

1

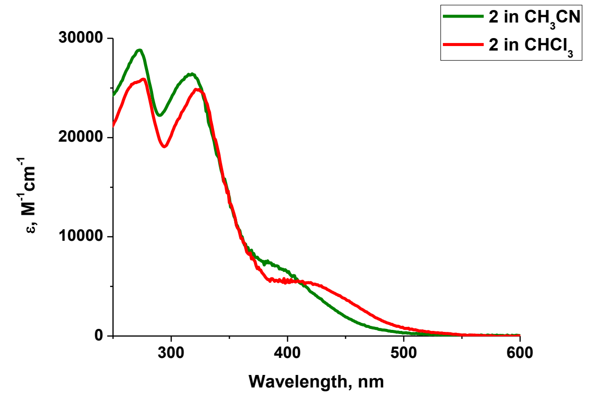

2

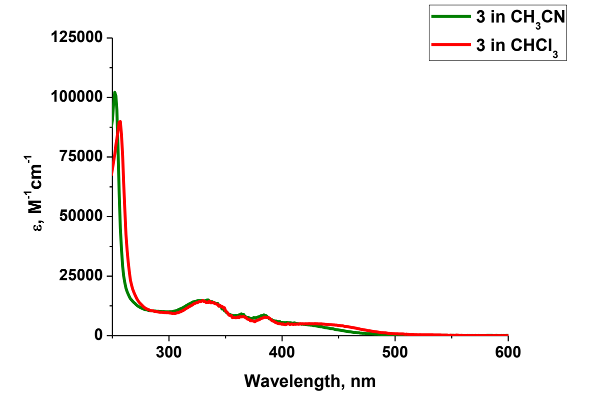

3

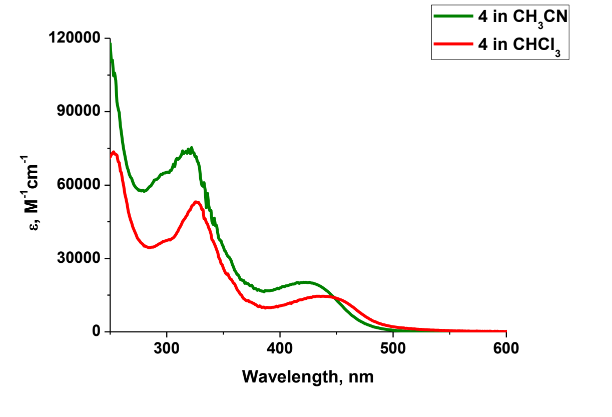

4

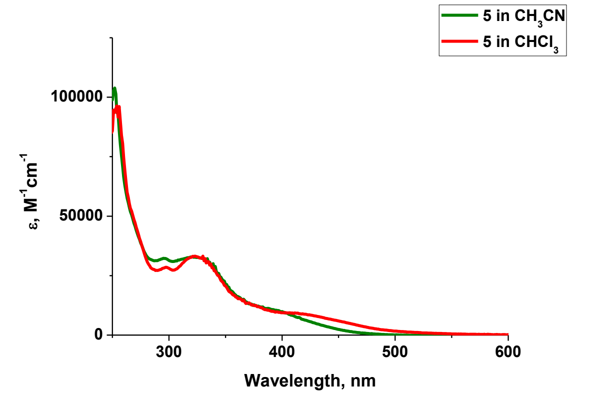

5

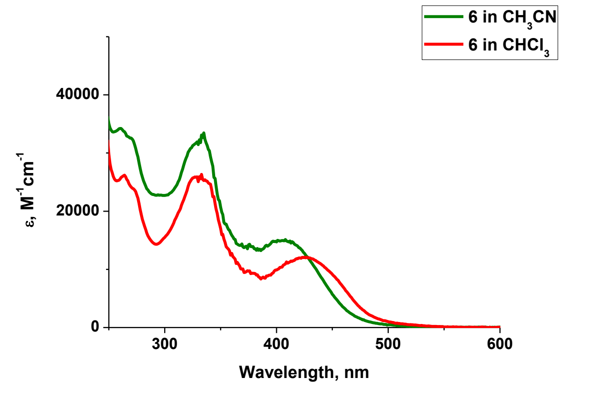

6

DFT and TDDFT calculations

Figure S12. Geometry optimization of **1**–**4** compared with structure determined by X-ray analysis

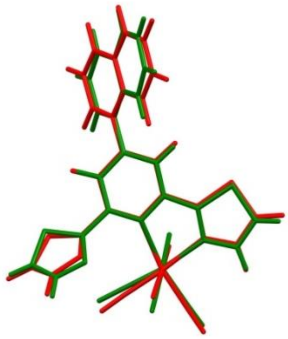	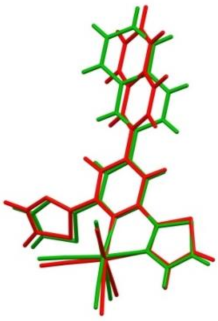
**1**	**2**
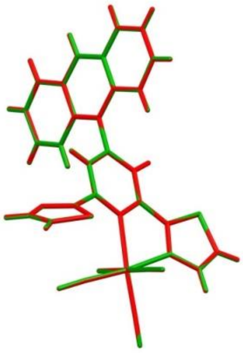	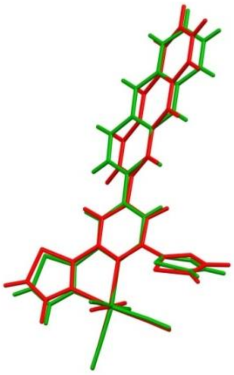
**3**	**4**

Table S9. Experimental and calculated dihedral angles [°] between the plane of polyaromatic group and central pyridine ring

Compound	Structure determined by X-Ray analysis	Theoretical geometry S_0 ($CHCl_3$)	Theoretical geometry T_1 ($CHCl_3$)
**1**	57.70	54.45	50.50
**2**	14.60	32.36	23.91
**3**	87.02	88.92	54.93
**4**	8.03	31.64	19.14
**5**	—	55.58	57.10
**6**	—	54.92	40.71

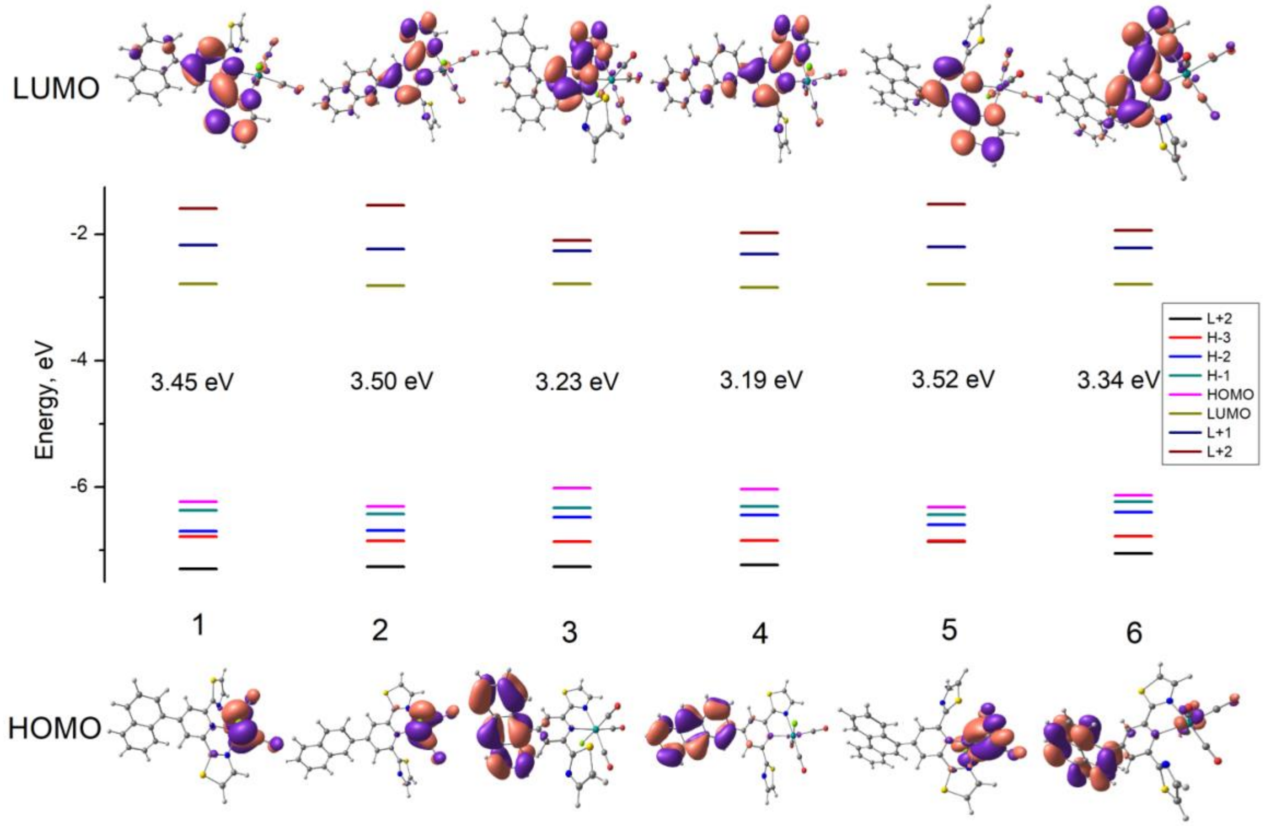

Figure S13. The partial molecular orbital energy level diagrams for **1**–**6**, along with plots of their frontier molecular orbitals

Table S10. Experimental (black line) and theoretical (blue line) absorption spectra and calculated transitions of **1** in chloroform (TD-DFT/PCM/PBE1PBE/def2-TZVPD/def2-TZVP)

1

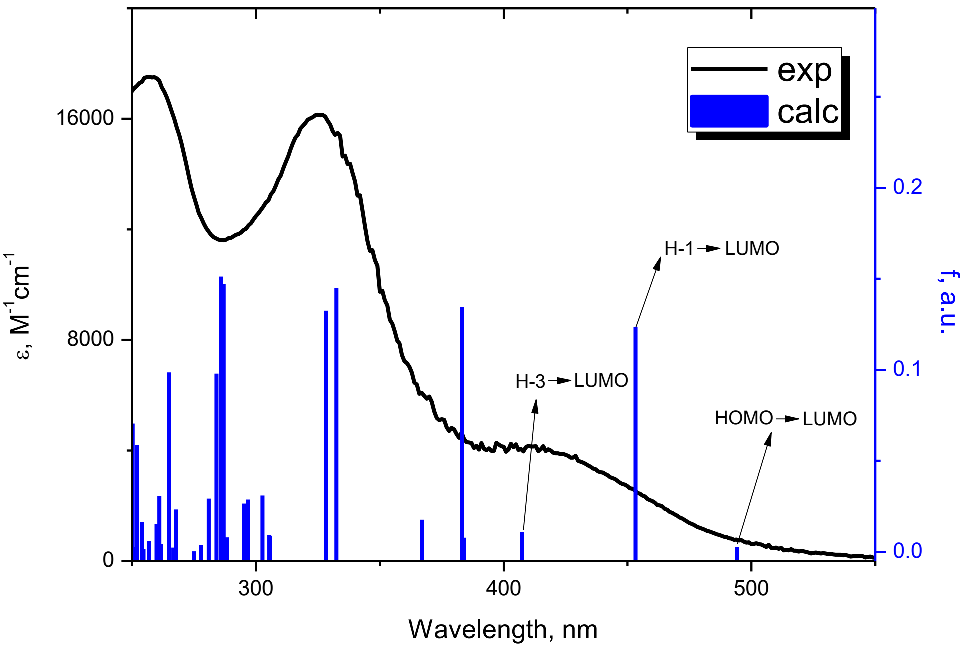

Exp	Calc	*f*	Transition	Character
415 (4.10)	494.1	0.0027	HOMO→LUMO (98%)	d_{Re} (46%), π^*_{CO} (25%), π_{Cl} (20%) → π^*_{dtpy} (76%), π^*_{R} (18%)
	453.2	0.1236	H-1→LUMO (96%)	d_{Re} (39%), π^*_{CO} (19%), π_{Cl} (18%) → π^*_{dtpy} (76%), π^*_{R} (18%)
	407.5	0.0108	H-3→LUMO (96%)	d_{Re} (56%), π^*_{CO} (27%) → π^*_{dtpy} (76%), π^*_{R} (18%)
326 (16.30)	383.1	0.1343	H-2→LUMO (94%)	π_{dtpy} (74%), π_{R} (17%) → π^*_{dtpy} (76%), π^*_{R} (18%)
	367.0	0.0177	H-1→L+1 (97%)	d_{Re} (39%), π^*_{CO} (19%), π_{Cl} (18%) → π^*_{dtpy} (67%), π^*_{R} (31%)
	332.5	0.145	H-4→LUMO (54%),<br>H-3→L+1 (14%),<br>H-2→L+1 (29%)	π_{dtpy} (89%) → π^*_{dtpy} (76%), π^*_{R} (18%);<br>d_{Re} (56%), π^*_{CO} (27%) → π^*_{dtpy} (67%), π^*_{R} (31%);<br>π_{dtpy} (74%), π_{R} (17%) → π^*_{dtpy} (67%), π^*_{R} (31%)
	328.4	0.1325	H-4→LUMO (40%),<br>H-3→L+1 (21%),<br>H-2→L+1 (35%)	π_{dtpy} (89%) → π^*_{dtpy} (76%), π^*_{R} (18%);<br>d_{Re} (56%), π^*_{CO} (27%) → π^*_{dtpy} (67%), π^*_{R} (31%);<br>π_{dtpy} (74%), π_{R} (17%) → π^*_{dtpy} (67%), π^*_{R} (31%)
258 (17.61)	287.0	0.1472	H-8→LUMO (57%),<br>H-6→LUMO (10%),<br>H-4→L+1 (16%)	π_{Cl} (50%), π_{dtpy} (31%) → π^*_{dtpy} (76%), π^*_{R} (18%);<br>π_{dtpy} (74%), π_{Cl} (18%) → π^*_{dtpy} (76%), π^*_{R} (18%);<br>π_{dtpy} (89%) → π^*_{dtpy} (67%), π^*_{R} (31%)
	285.9	0.1513	H-8→LUMO (13%),	π_{Cl} (50%), π_{dtpy} (31%) → π^*_{dtpy} (76%), π^*_{R} (18%);

			H-4→L+1 (16%),<br>H-2→L+2 (22%),<br>H-1→L+3 (14%),<br>HOMO→L+5 (10%)	π_{dtpy} (89%) → π^{*}_{dtpy} (67%), π^{*}_{R} (31%);<br>π_{dtpy} (74%), π_{R} (17%) →π^{*}_{R} (63%), π^{*}_{dtpy} (35%);<br>d_{Re} (39%), π^{*}_{CO} (19%), π_{Cl} (18%) →π^{*}_{dtpy} (71%), π^{*}_{R} (20%);<br>d_{Re} (46%), π^{*}_{CO} (25%), π_{Cl} (20%) → π^{*}_{dtpy} (44%), π^{*}_{CO} (20%), d_{Re} (15%), π_{Cl} (13%)
	284.1	0.0979	H-4→L+1 (28%),<br>HOMO→L+5 (40%)	π_{dtpy} (89%) → π^{*}_{dtpy} (67%), π^{*}_{R} (31%);<br>d_{Re} (46%), π^{*}_{CO} (25%), π_{Cl} (20%) → π^{*}_{dtpy} (44%), π_{CO} (20%), d_{Re} (15%), π^{*}_{Cl} (13%)
	264.917<br>828705	0.0986	H-6→L+1 (70%)	π_{dtpy} (74%), π_{Cl} (18%)→ π^{*}_{dtpy} (67%), π^{*}_{R} (31%);

Table S11. Experimental (black line) and theoretical (blue line) absorption spectra and calculated transitions of **2** in chloroform (TD-DFT/PCM/PBE1PBE/def2-TZVPD/def2-TZVP)

2

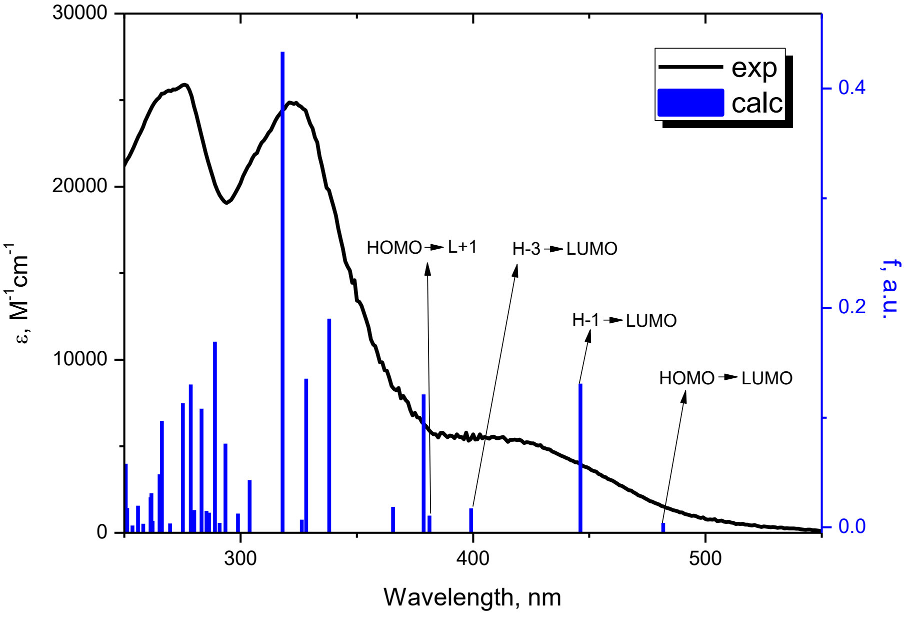

Exp	Calc	*f*	Transition	Character
416 (5.53)	481.8	0.004	HOMO→LUMO (98%)	d_{Re} (45%), π^*_{CO} (25%), π_{Cl} (19%), π_{dtpy} (10%) → π^*_{dtpy} (63%), π^*_{R} (24%)
	446.1	0.131	H-1→LUMO (96%)	d_{Re} (38%), π^*_{CO} (19%), π_{Cl} (19%), π_{dtpy} (19%) → π^*_{dtpy} (63%), π^*_{R} (24%)
	399.2	0.0173	H-3→LUMO (92%)	d_{Re} (55%), π^*_{CO} (25%), π_{dtpy} (16%) → π^*_{dtpy} (63%), π^*_{R} (24%)
	381.2	0.0107	HOMO→L+1 (96%)	d_{Re} (45%), π^*_{CO} (25%), π_{Cl} (19%), π_{dtpy} (10%)→ π^*_{dtpy} (63%), π^*_{R} (35%)
324 (24.93)	378.7	0.1211	H-2→LUMO (92%)	π_{R} (82%), π_{dtpy} (10%) → π^*_{dtpy} (63%), π^*_{R} (24%)
	365.6	0.0187	H-1→L+1 (96%)	d_{Re} (38%), π^*_{CO} (19%), π_{Cl} (19%), π_{dtpy} (19%) → π^*_{dtpy} (63%), π^*_{R} (35%)
	338.2	0.1903	H-4→LUMO (70%),<br>H-2→L+1 (13%)	π_{dtpy} (78%) → → π^*_{dtpy} (63%), π^*_{R} (24%);<br>π_{R} (82%), π_{dtpy} (10%) → π^*_{dtpy} (63%), π^*_{R} (35%)
	328.3	0.1356	H-4→LUMO (20%),<br>H-3→L+1 (10%),<br>H-2→L+1 (63%)	π_{dtpy} (78%) → π^*_{dtpy} (63%), π^*_{R} (24%);<br>d_{Re} (55%), π^*_{CO} (25%), π_{dtpy} (16%) → π^*_{dtpy} (63%), π^*_{R} (35%);<br>π_{R} (82%), π_{dtpy} (10%) → π^*_{dtpy} (63%), π^*_{R} (35%)
	318.0	0.4336	H-5→LUMO (77%),	π_{R} (75%), π_{dtpy} (16%) → π^*_{dtpy} (63%), π^*_{R} (24%);

			H-2→L+1 (11%)	π_R (82%), π_{dtpy} (10%) → π^*_{dtpy} (63%), π^*_R (35%)
276 (25.91)	289.0	0.1693	H-7→LUMO (32%),<br>H-4→L+1 (44%)	π_{Cl} (50%), π_{dtpy} (31%) → π^*_{dtpy} (63%), π^*_R (24%);<br>π_{dtpy} (78%) → π^*_{dtpy} (63%), π^*_R (35%)
	278.6	0.1302	H-8→LUMO (75%),<br>H-5→L+1 (13%)	π_{Cl} (45%), π_{dtpy} (29%) → π^*_{dtpy} (63%), π^*_R (24%);<br>π_R (75%), π_{dtpy} (16%) → π^*_{dtpy} (63%), π^*_R (35%)
	275.2	0.1133	H-5→L+1 (14%),<br>H-2→L+2 (67%)	π_R (75%), π_{dtpy} (16%) → π^*_{dtpy} (63%), π^*_R (35%);<br>π_R (82%), π_{dtpy} (10%) → π^*_R (60%), π^*_{dtpy} (38%)
267 (sh 25.43)	266.1	0.097	H-6→L+1 (29%),<br>H-5→L+2 (10%),<br>H-2→L+3 (12%),<br>H-1→L+3 (13%)	π_{dtpy} (67%), π_{Cl} (24%) → π^*_{dtpy} (63%), π^*_R (35%);<br>π_R (75%), π_{dtpy} (16%) → π^*_R (60%), π^*_{dtpy} (38%);<br>π_R (82%), π_{dtpy} (10%) → π^*_{dtpy} (73%), π^*_{dtpy} (22%);<br>d_{Re} (38%), π^*_{CO} (19%), π_{Cl} (19%), π_{dtpy} (19%) → π^*_{dtpy} (73%), π^*_{dtpy} (22%)

Table S12. Experimental (black line) and theoretical (blue line) absorption spectra and calculated transitions of **3** in chloroform (TD-DFT/PCM/PBE1PBE/def2-TZVPD/def2-TZVP)

3

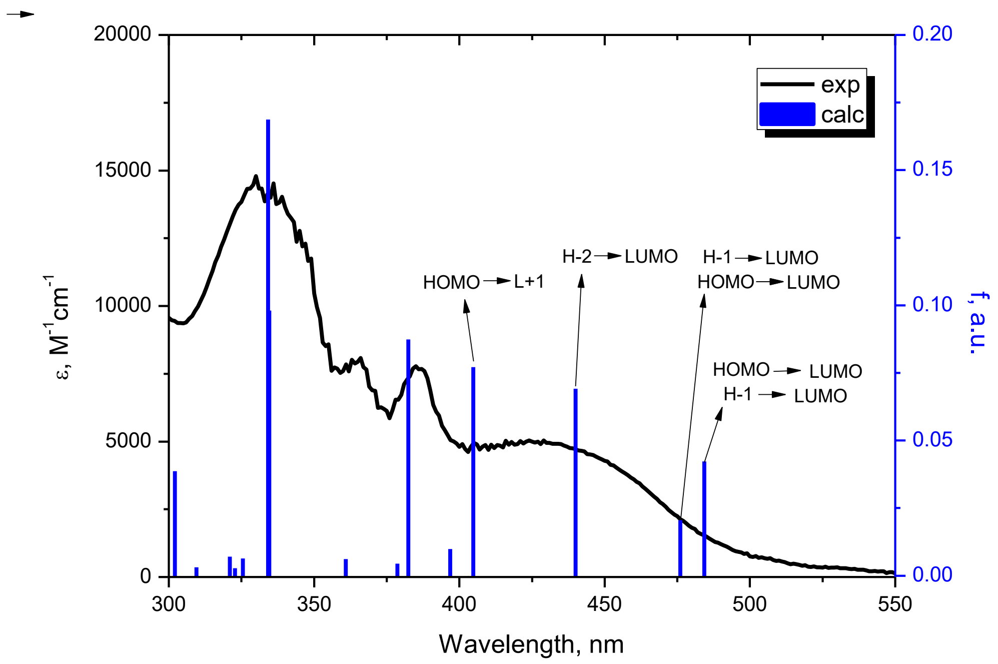

Exp	Calc	*f*	Transition	Character
430 (5.04)	484.3	0.0423	HOMO→LUMO (85%); H-1→LUMO (12%),	π_R (77%), π_{dtpy} (12%) → π^*_R (52%), π^*_{dtpy} (46%), d_{Re} (40%), π^*_{CO} (22%), π_{Cl} (17%), π_{dtpy} (13%) → π^*_R (52%), π^*_{dtpy} (46%)
	476.0	0.0205	H-1→LUMO (85%), HOMO→LUMO (13%)	d_{Re} (40%), π^*_{CO} (22%), π_{Cl} (17%), π_{dtpy} (13%) → π^*_R (52%), π^*_{dtpy} (46%); π_R (77%), π_{dtpy} (12%) → π^*_R (52%), π^*_{dtpy} (46%),
	439.9	0.0692	H-2→LUMO (96%)	d_{Re} (32%), π^*_{CO} (16%), π_{Cl} (17%), π_{dtpy} (18%), π_R (19%) → π^*_R (52%), π^*_{dtpy} (46%)
	404.8	0.0771	HOMO→L+1 (90%)	π_R (77%), π_{dtpy} (12%) → π^*_{dtpy} (72%), π^*_R (12%)
	396.8	0.0099	H-3→LUMO (96%)	d_{Re} (54%), π^*_{CO} (25%), π_{dtpy} (18%) → π^*_R (52%), π^*_{dtpy} (46%)
386 (7.85)	382.4	0.0874	HOMO→L+2 (90%)	π_R (77%), π_{dtpy} (12%) → π^*_{dtpy} (58%), π^*_R (41%)
	378.6	0.0044	H-1→L+1 (67%), H-1→L+2 (31%)	d_{Re} (40%), π^*_{CO} (22%), π_{Cl} (17%), π_{dtpy} (13%) → π^*_{dtpy} (72%), π^*_R (12%), d_{Re} (40%), π^*_{CO} (22%), π_{Cl} (17%), π_{dtpy} (13%) → π^*_{dtpy} (58%), π^*_R (41%)
365 (8.07)	360.9	0.0061	H-2→L+1 (69%),	d_{Re} (32%), π^*_{CO} (16%), π_{Cl} (17%), π_{dtpy} (18%), π_R

			H-2→L+2 (29%)	(19%) → π^*_{dtpy} (72%), π^*_R (12%),<br>d_{Re} (32%), π^*_{CO} (16%), π_{Cl} (17%), π_{dtpy} (18%), π_R (19%) → π^*_{dtpy} (58%), π^*_R (41%)
333 (14.43)	334.6	0.0981	H-4→LUMO (32%),<br>H-1→L+1 (19%),<br>H-1→L+2 (41%)	π_{dtpy} (68%), π_R (18%) → π^*_R (52%), π^*_{dtpy} (46%)<br>d_{Re} (40%), π^*_{CO} (22%), π_{Cl} (17%), π_{dtpy} (13%) → π^*_{dtpy} (72%), π^*_R (12%)<br>d_{Re} (40%), π^*_{CO} (22%), π_{Cl} (17%), π_{dtpy} (13%) → π^*_{dtpy} (58%), π^*_R (41%)
	334.2	0.1687	H-4→LUMO (50%),<br>H-1→L+1 (12%),<br>H-1→L+2 (26%)	π_{dtpy} (68%), π_R (18%)→ π^*_R (52%), π^*_{dtpy} (46%)<br>d_{Re} (40%), π^*_{CO} (22%), π_{Cl} (17%), π_{dtpy} (13%) → π^*_{dtpy} (72%), π^*_R (12%)<br>d_{Re} (40%), π^*_{CO} (22%), π_{Cl} (17%), π_{dtpy} (13%) → π^*_{dtpy} (58%), π^*_R (41%)

Table S13. Experimental (black line) and theoretical (blue line) absorption spectra and calculated transitions of **4** in chloroform (TD-DFT/PCM/PBE1PBE/def2-TZVPD/def2-TZVP)

4

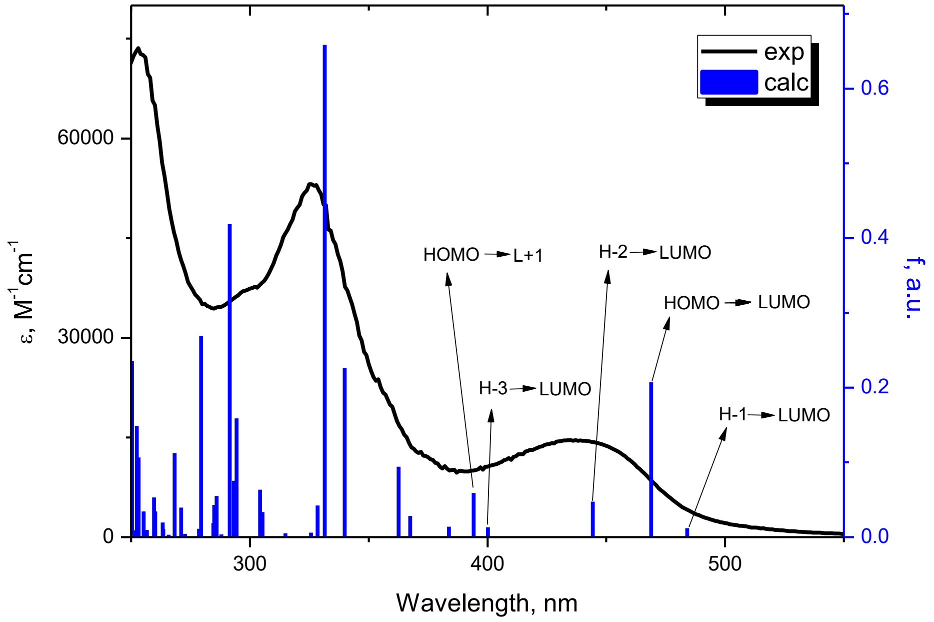

Exp	Calc	*f*	Transition	Character
438 (14.61)	484.0	0.0118	H-1→LUMO (89%)	d_{Re} (44%), π^*_{CO} (24%), π_{Cl} (19%), π_{dtpy} (11%) → π^*_{dtpy} (59%), π^*_{R} (30%)
	468.9	0.2071	HOMO→LUMO (87%)	π_R (88%) → π^*_{dtpy} (59%), π^*_{R} (30%)
	444.3	0.0475	H-2→LUMO (89%)	→ π^*_{dtpy} (59%), π^*_{R} (30%)
	400.2	0.0132	H-3→LUMO (93%)	→ π^*_{dtpy} (59%), π^*_{R} (30%)
	394.1	0.0591	HOMO→L+1 (96%)	π_R (88%) → π^*_{dtpy} (58%), π^*_{R} (39%)
373 (sh 12.53)	383.8	0.0139	H-1→L+1 (84%),<br>H-1→L+2 (12%)	d_{Re} (44%), π^*_{CO} (24%), π_{Cl} (19%), π_{dtpy} (11%) → π^*_{dtpy} (58%), π^*_{R} (39%),<br>d_{Re} (44%), π^*_{CO} (24%), π_{Cl} (19%), π_{dtpy} (11%) →π^*_{R} (52%), π^*_{dtpy} (47%)
	367.5	0.0285	H-2→L+1 (82%),<br>H-2→L+2 (10%)	d_{Re} (38%), π^*_{CO} (19%), π_{Cl} (21%), π_{dtpy} (18%) → π^*_{dtpy} (58%), π^*_{R} (39%),<br>d_{Re} (38%), π^*_{CO} (19%), π_{Cl} (21%), π_{dtpy} (18%) → π^*_{R} (52%), π^*_{dtpy} (47%)
355 (sh 22.47)	362.6	0.0941	HOMO→L+2 (84%)	π_R (88%) → π^*_{R} (52%), π^*_{dtpy} (47%)
326 (53.08)	339.9	0.2262	H-5→LUMO (34%),<br>H-4→LUMO (53%)	π_{dtpy} (45%), π_R (44%) → π^*_{dtpy} (59%), π^*_{R} (30%),<br>π_{dtpy} (53%), π_R (37%) → π^*_{dtpy} (59%), π^*_{R} (30%)
	331.5	0.6585	H-5→LUMO (48%),<br>H-4→LUMO (18%),	π_{dtpy} (45%), π_R (44%) → π^*_{dtpy} (59%), π^*_{R} (30%),<br>π_{dtpy} (53%), π_R (37%) → π^*_{dtpy} (59%), π^*_{R} (30%)

			H-3→L+1 (10%)	d_{Re} (54%), π^*_{CO} (25%), π_{dtpy} (18%) → π^*_{dtpy} (58%), π^*_{R} (39%)
300 (37.59)	294.3	0.159	H-8→LUMO (44%),<br>H-7→LUMO (11%)	π_{Cl} (48%), π_{dtpy} (32%) → π^*_{dtpy} (59%), π^*_{R} (30%),<br>π_{R} (74%), π_{dtpy} (15%) → π^*_{dtpy} (59%), π^*_{R} (30%)
	291.5	0.4185	H-8→LUMO (16%),<br>H-7→LUMO (10%),<br>H-4→L+1 (46%)	π_{Cl} (48%), π_{dtpy} (32%) → π^*_{dtpy} (59%), π^*_{R} (30%),<br>π_{R} (74%), π_{dtpy} (15%) → π^*_{dtpy} (59%), π^*_{R} (30%),<br>π_{dtpy} (53%), π_{R} (37%) → π^*_{dtpy} (58%), π^*_{R} (39%)

Table S14. Experimental (black line) and theoretical (blue line) absorption spectra and calculated transitions of **5** in chloroform (TD-DFT/PCM/PBE1PBE/def2-TZVPD/def2-TZVP)

5

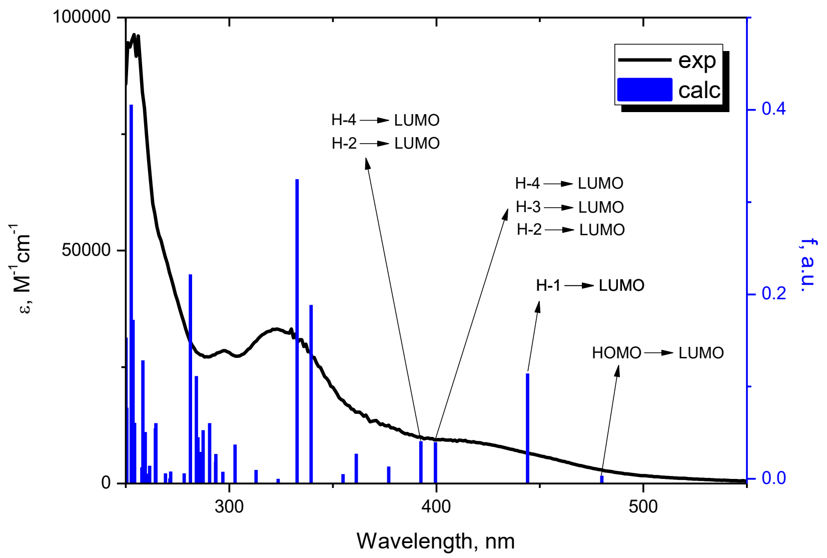

Exp	Calc	*f*	Transition	Character
413 (9.33)	479.9	0.0035	HOMO→LUMO (98%)	d_{Re} (44%), π^*_{CO} (24%), π_{Cl} (19%), π_{dtpy} (10%) → π^*_{dtpy} (79%)
	444.0	0.1142	H-1→LUMO (93%)	d_{Re} (34%), π_R (18%), π^*_{CO} (17%), π_{Cl} (17%), π_{dtpy} (14%) → π^*_{dtpy} (79%)
	399.6	0.0398	H-4→LUMO (46%),<br>H-3→LUMO (22%),<br>H-2→LUMO (27%)	d_{Re} (39%), π_R (24%), π^*_{CO} (18%), π_{dtpy} (16%) → π^*_{dtpy} (79%);<br>π_R (64%), d_{Re} (18%) → π^*_{dtpy} (79%);<br>π_R (62%), π_{dtpy} (23%) → π^*_{dtpy} (79%)
	392.5	0.0409	H-4→LUMO (23%),<br>H-2→LUMO (67%)	d_{Re} (39%), π_R (24%), π^*_{CO} (18%), π_{dtpy} (16%) → π^*_{dtpy} (79%);<br>π_R (62%), π_{dtpy} (23%) → π^*_{dtpy} (79%)
323 (33.09)	339.6	0.1886	H-2→L+1 (85%)	π_R (62%), π_{dtpy} (23%) → π^*_{dtpy} (80%), π^*_R (19%)
	332.7	0.3249	H-5→LUMO (83%),<br>H-2→L+1 (10%)	π_{dtpy} (82%) → π^*_{dtpy} (79%)<br>π_R (62%), π_{dtpy} (23%) → π^*_{dtpy} (80%), π^*_R (19%)
297 (28.67)	302.8	0.0373	H-6→LUMO (89%)	π_{dtpy} (66%), π_{Cl} (24%) → π^*_{dtpy} (79%)
	290.5	0.0606	H-1→L+2 (40%),<br>H-1→L+4 (11%),<br>H-1→L+5 (13%)	d_{Re} (34%), π_R (18%), π^*_{CO} (17%), π_{Cl} (17%), π_{dtpy} (14%) → π^*_R (75%), π^*_{dtpy} (24%);<br>d_{Re} (34%), π_R (18%), π^*_{CO} (17%), π_{Cl} (17%), π_{dtpy} (14%) → π^*_{dtpy} (80%), π^*_R (13%);<br>d_{Re} (34%), π_R (18%), π^*_{CO} (17%), π_{Cl} (17%), π_{dtpy} (14%)

				→ π^{*}_{dtpy} (34%), π_{CO} (28%), d_{Re} (25%), π^{*}_{R} (11%)
	284.2	0.1116	H-5→L+1 (35%),<br>HOMO→L+2 (17%),<br>HOMO→L+5 (12%)	π_{dtpy} (82%) → π^{*}_{dtpy} (80%), π^{*}_{R} (19%)<br>d_{Re} (44%), π^{*}_{CO} (24%), π_{Cl} (19%), π_{dtpy} (10%) → π^{*}_{R} (75%), π^{*}_{dtpy} (24%);<br>d_{Re} (44%), π^{*}_{CO} (24%), π_{Cl} (19%), π_{dtpy} (10%) → π^{*}_{dtpy} (34%), π_{CO} (28%), d_{Re} (25%), π^{*}_{R} (11%)
	281.2	0.2217	H-9→LUMO (33%),<br>H-8→LUMO (16%),<br>H-7→LUMO (15%),<br>H-5→L+1 (21%)	π_{Cl} (41%), π_{dtpy} (30%) → π^{*}_{dtpy} (79%);<br>π_{Cl} (40%), π_{dtpy} (26%), π_{R} (20%) → π^{*}_{dtpy} (79%);<br>π_{Cl} (24%), π_{dtpy} (15%), π_{R} (53%) → π^{*}_{dtpy} (79%)<br>π_{dtpy} (82%) → π^{*}_{dtpy} (80%), π^{*}_{R} (19%)
254 (96.49)	258.2	0.1284	H-4→L+2 (26%),<br>H-3→L+2 (21%),<br>H-2→L+3 (38%)	d_{Re} (39%), π_{R} (24%), π^{*}_{CO} (18%), π_{dtpy} (16%) → π^{*}_{R} (75%), π^{*}_{dtpy} (24%);<br>π_{R} (64%), d_{Re} (18%) → π^{*}_{R} (75%), π^{*}_{dtpy} (24%);<br>π_{R} (62%), π_{dtpy} (23%) → π^{*}_{R} (88%)
	253.4	0.1724	H-8→L+1 (25%),<br>H-7→L+1 (36%),<br>H-3→L+3 (11%)	π_{Cl} (40%), π_{dtpy} (26%), π_{R} (20%) → π^{*}_{dtpy} (80%), π^{*}_{R} (19%);<br>π_{Cl} (24%), π_{dtpy} (15%), π_{R} (53%) → π^{*}_{dtpy} (80%), π^{*}_{R} (19%);<br>π_{R} (64%), d_{Re} (18%) → π^{*}_{R} (88%)
	252.6	0.4055	H-4→L+3 (10%),<br>H-3→L+3 (36%)	d_{Re} (39%), π_{R} (24%), π^{*}_{CO} (18%), π_{dtpy} (16%) → π^{*}_{R} (88%);<br>π_{R} (64%), d_{Re} (18%) → π^{*}_{R} (88%)

Table S15. Experimental (black line) and theoretical (blue line) absorption spectra and calculated transitions of **6** in chloroform (TD-DFT/PCM/PBE1PBE/def2-TZVPD/def2-TZVP)

6

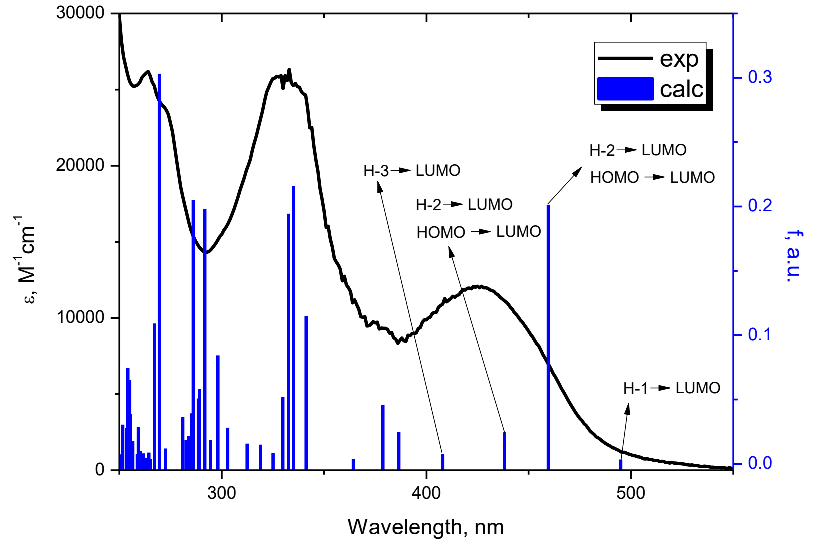

Exp	Calc	*f*	Transition	Character
426 (12.08)	495.0	0.0033	H-1→LUMO (91%)	d_{Re} (46%), π^*_{CO} (24%), π_{Cl} (19%) → π^*_{dtpy} (67%), π^*_{R} (24%)
	459.6	0.2013	H-2→LUMO (40%), HOMO→LUMO (51%)	d_{Re} (35%), π^*_{CO} (18%), π_{Cl} (18%), π_{dtpy} (18%), π_{R} (10%) → π^*_{dtpy} (67%), π^*_{R} (24%), π_{R} (84%) → π^*_{dtpy} (67%), π^*_{R} (24%)
	438.1	0.0244	H-2→LUMO (56%), HOMO→LUMO (42%)	d_{Re} (35%), π^*_{CO} (18%), π_{Cl} (18%), π_{dtpy} (18%), π_{R} (10%) → π^*_{dtpy} (67%), π^*_{R} (24%), π_{R} (84%) → π^*_{dtpy} (67%), π^*_{R} (24%)
	408.0	0.0076	H-3→LUMO (97%)	d_{Re} (56%), π^*_{CO} (27%), π_{dtpy} (14%) → π^*_{dtpy} (67%), π^*_{R} (24%)
378 (sh 9.53)	386.5	0.0247	H-1→L+1 (80%), HOMO→L+1 (10%)	d_{Re} (46%), π^*_{CO} (24%), π_{Cl} (19%) → π^*_{R} (50%), π^*_{dtpy} (49%), π_{R} (84%) → π^*_{R} (50%), π^*_{dtpy} (49%)
	378.8	0.0456	HOMO→L+1 (75%)	π_{R} (84%) → π^*_{R} (50%), π^*_{dtpy} (49%)
	364.3	0.0035	H-2→L+1 (84%)	d_{Re} (35%), π^*_{CO} (18%), π_{Cl} (18%), π_{dtpy} (18%), π_{R} (10%) → π^*_{R} (50%), π^*_{dtpy} (49%)
331 (25.94)	341.3	0.1148	H-4→LUMO (64%), HOMO→L+2 (20%)	π_{R} (95%) → π^*_{dtpy} (67%), π^*_{R} (24%). π_{R} (84%) → π^*_{R} (56%), π^*_{dtpy} (44%)
	335.1	0.2158	H-4→LUMO (16%), HOMO→L+2 (69%)	π_{R} (95%) → π^*_{dtpy} (67%), π^*_{R} (24%) π_{R} (84%) → π^*_{R} (56%), π^*_{dtpy} (44%)
	332.6	0.1944	H-5→LUMO (61%),	π_{dtpy} (90%) → π^*_{dtpy} (67%), π^*_{R} (24%),

			H-3→L+1 (31%)	d_{Re} (56%), π^*_{CO} (27%), π_{dtpy} (14%) → π^*_R (50%), π^*_{dtpy} (49%)
272 (sh 23.78)	291.7	0.1981	H-4→L+1 (67%),<br>HOMO→L+3 (11%)	π_R (95%) → π^*_R (50%), π^*_{dtpy} (49%),<br>π_R (84%) → π^*_R (62%), π^*_{dtpy} (36%)
	286.1	0.2051	H-5→L+1 (46%)	π_{dtpy} (90%) → π^*_R (50%), π^*_{dtpy} (49%),
264 (26.24)	269.6	0.3031	H-4→L+2 (47%),<br>HOMO→L+3 (24%)	π_R (95%) → π^*_R (56%), π^*_{dtpy} (44%),<br>π_R (84%) → π^*_R (62%), π^*_{dtpy} (36%)
	267.2	0.1092	H-6→L+1 (73%)	π_{dtpy} (45%), π_R (34%) → π^*_R (50%), π^*_{dtpy} (49%)

Stability and photostability studies

Figure S14. Stability (left panels) and photostability (right panels) of **1**–**6** in $CHCl_3$

Stability	Photostability

1

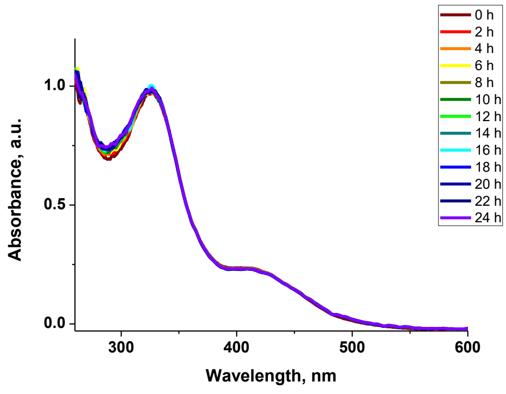

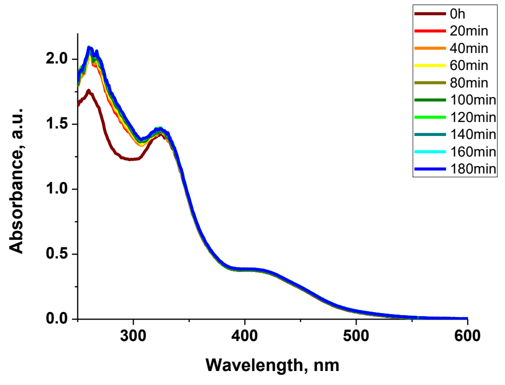

2

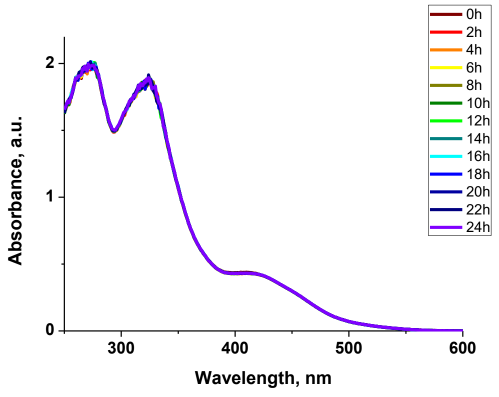

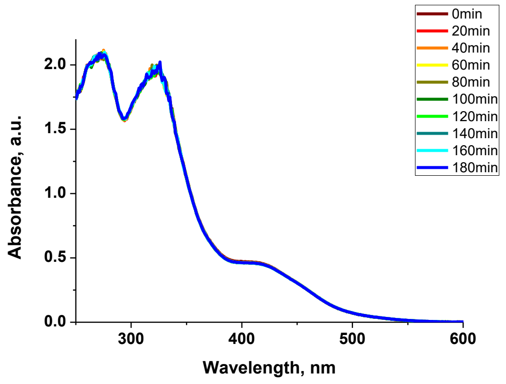

3

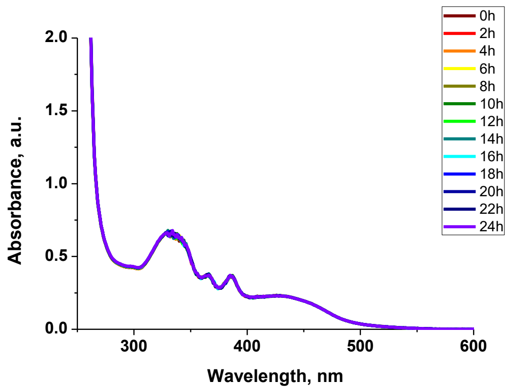

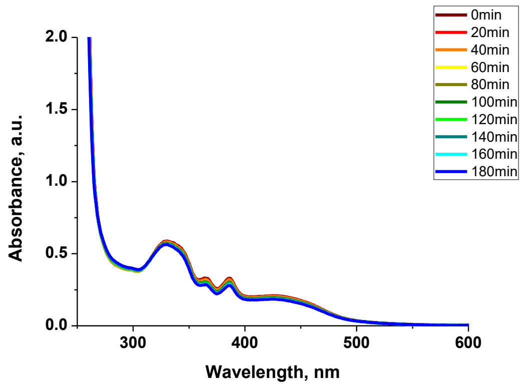

4

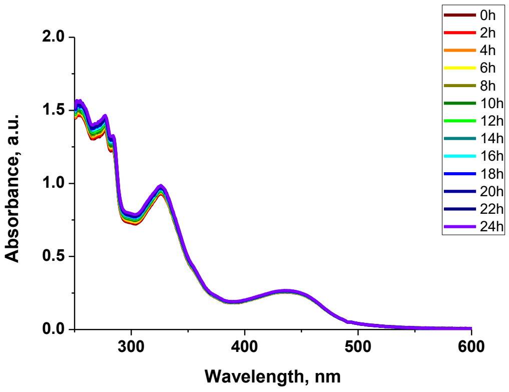
Absorbance, a.u.
Wavelength, nm
0.0
0.5
1.0
1.5
2.0
300
400
500
600
0h
2h
4h
6h
8h
10h
12h
14h
16h
18h
20h
22h
24h

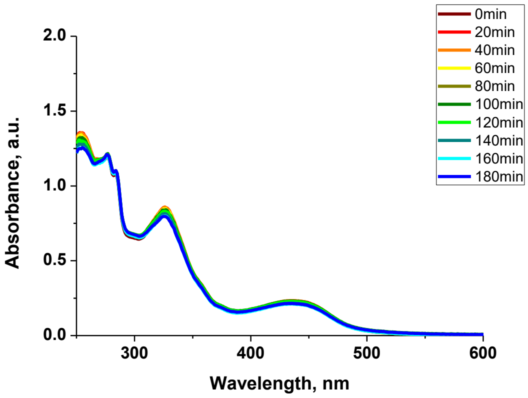
Absorbance, a.u.
Wavelength, nm
0.0
0.5
1.0
1.5
2.0
300
400
500
600
0min
20min
40min
60min
80min
100min
120min
140min
160min
180min

5

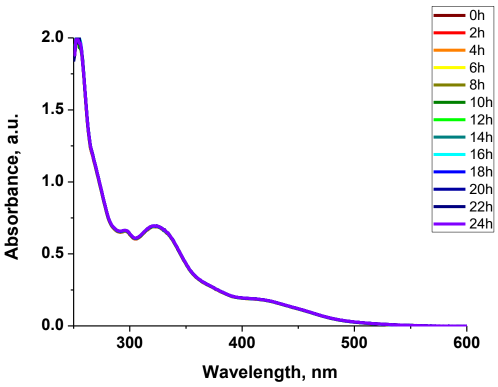
Absorbance, a.u.
Wavelength, nm
0.0
0.5
1.0
1.5
2.0
300
400
500
600
0h
2h
4h
6h
8h
10h
12h
14h
16h
18h
20h
22h
24h

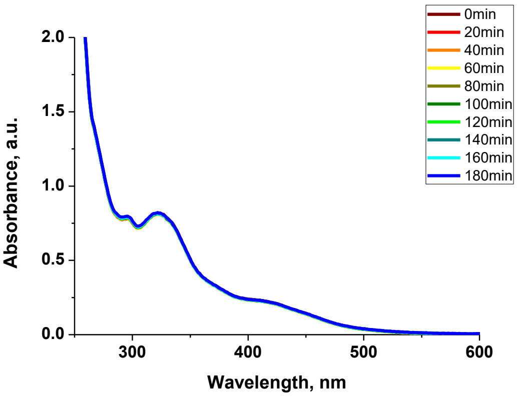
Absorbance, a.u.
Wavelength, nm
0.0
0.5
1.0
1.5
2.0
300
400
500
600
0min
20min
40min
60min
80min
100min
120min
140min
160min
180min

6

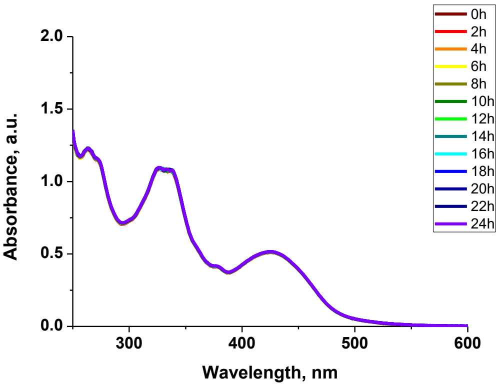
Absorbance, a.u.
Wavelength, nm
0.0
0.5
1.0
1.5
2.0
300
400
500
600
0h
2h
4h
6h
8h
10h
12h
14h
16h
18h
20h
22h
24h

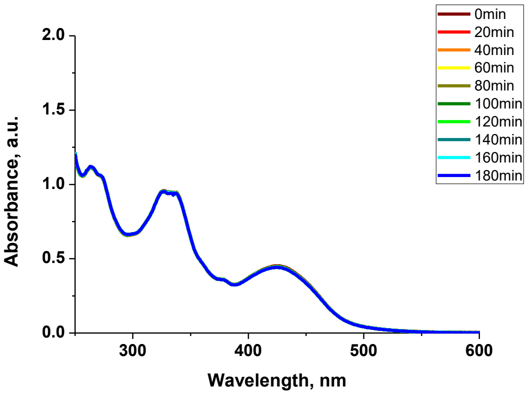
Absorbance, a.u.
Wavelength, nm
0.0
0.5
1.0
1.5
2.0
300
400
500
600
0min
20min
40min
60min
80min
100min
120min
140min
160min
180min

Figure S15. Stability (left panels) and photo-stability (right panel) of **1** in CH_3CN

CH_3CN	DMF
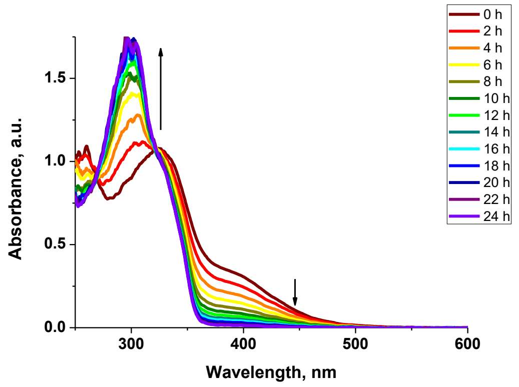	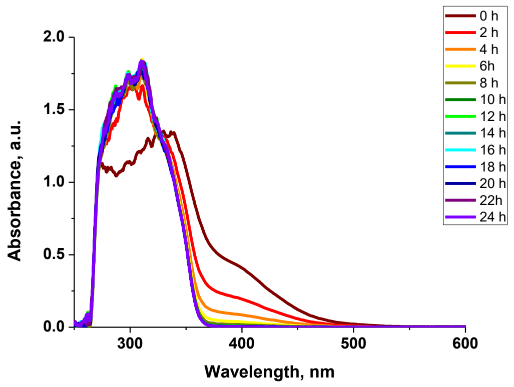

Photoluminescence properties

Figure S16. Normalized emission spectra of **1**–**6** in 77K

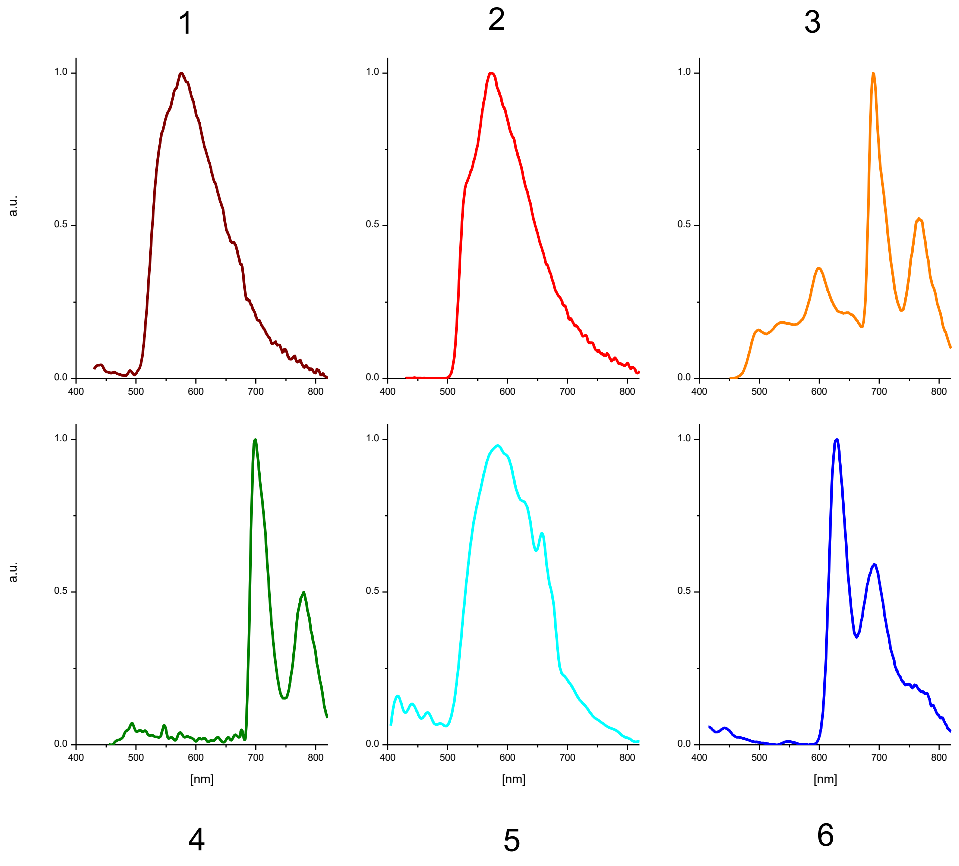

Figure S17. Decay curves of **1–6** in 77K

<table>
<tr><td colspan="4">1</td><td colspan="4">2</td></tr>
<tr><td colspan="4">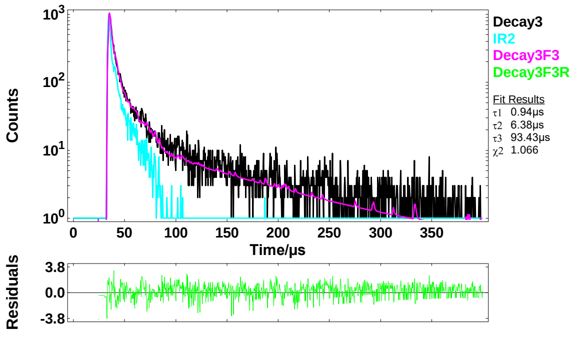
</td><td colspan="4">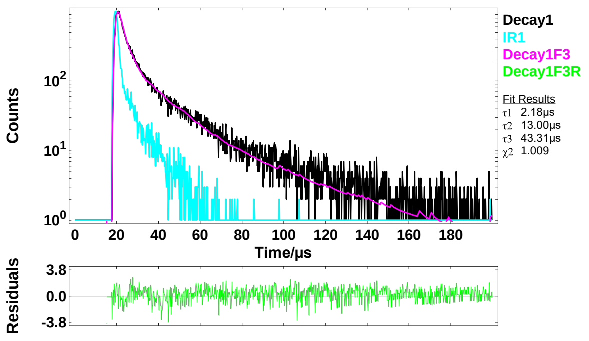
</td></tr>
<tr><td>τ</td><td>0.94 μs</td><td>6.38 μs</td><td>93.43 μs</td><td>τ</td><td>2.18 μs</td><td>13.00 μs</td><td>43.31μs</td></tr>
<tr><td>% I</td><td>78.84</td><td>4.27</td><td>16.89</td><td>% I</td><td>63.55</td><td>22.63</td><td>13.83</td></tr>
</table>

3

599 nm

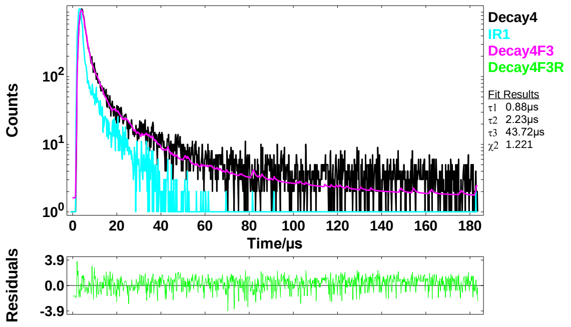

τ	0.88 µs	2.23 µs	43.70µs
%I	59.73	32.17	8.09

691 nm

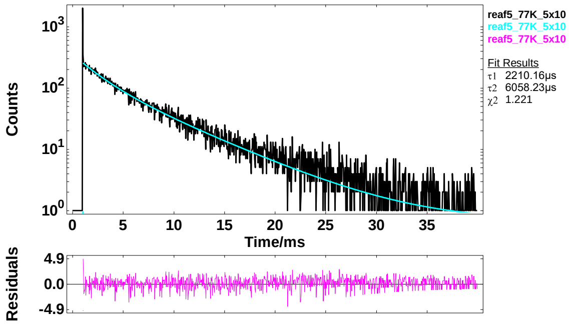

τ	2.21 ms	6.06 ms
%I	26.31	73.69

4

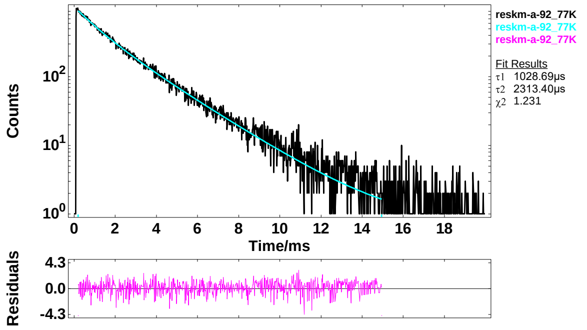

τ	1028.69 µs	2313.40 µs
%I	24.64	75.36

5			6		
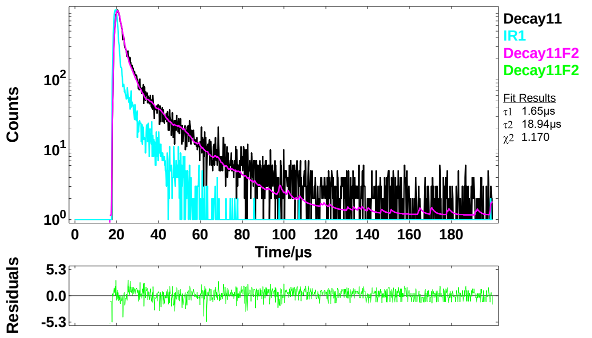			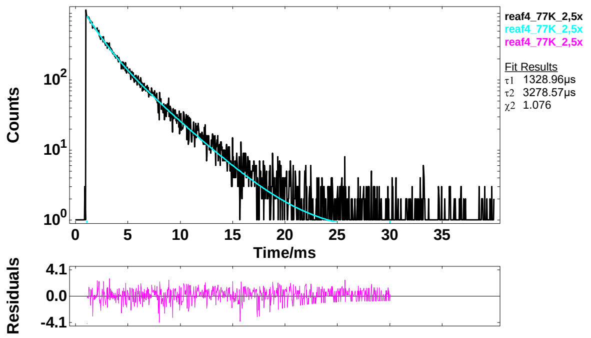		
τ	1.65 μs	18.94 μs	τ	1.33 ms	3.28 ms
%I	83.24	16.76	%I	33.47	66.53

Figure S18. Comparison of normalized emission spectra of **1**–**6** at 77K and at room temperature

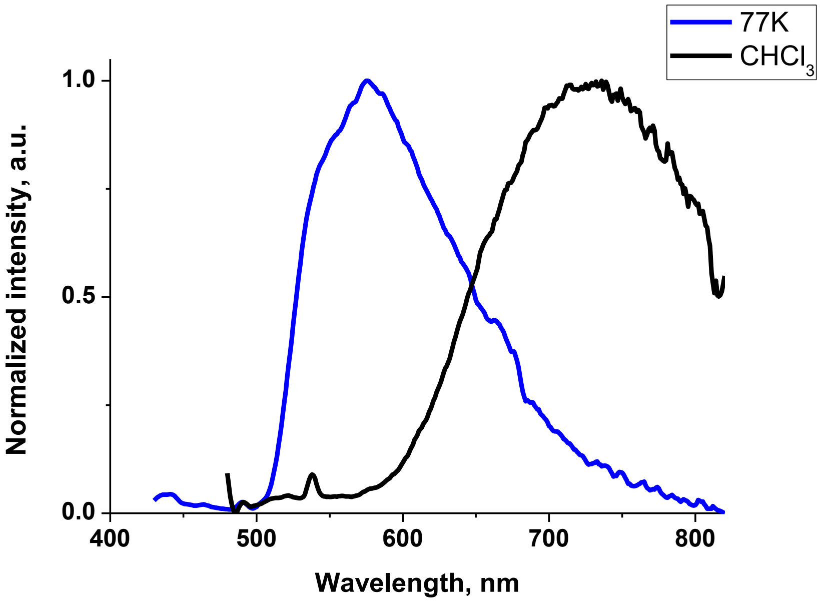

1

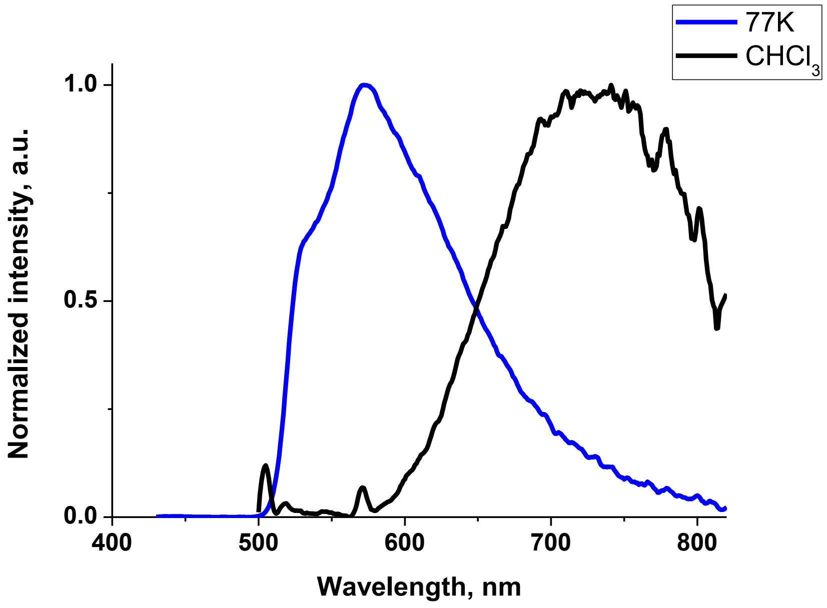
77K
CHCl3
1.0
0.5
0.0
Normalized intensity, a.u.
400
500
600
700
800
Wavelength, nm

2

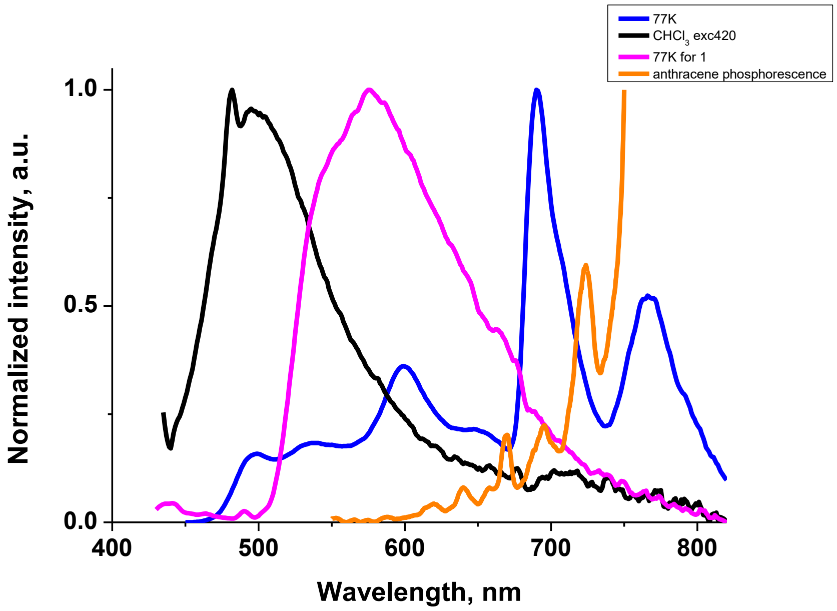
77K
CHCl3 exc420
77K for 1
anthracene phosphorescence
1.0
0.5
0.0
Normalized intensity, a.u.
400
500
600
700
800
Wavelength, nm

3

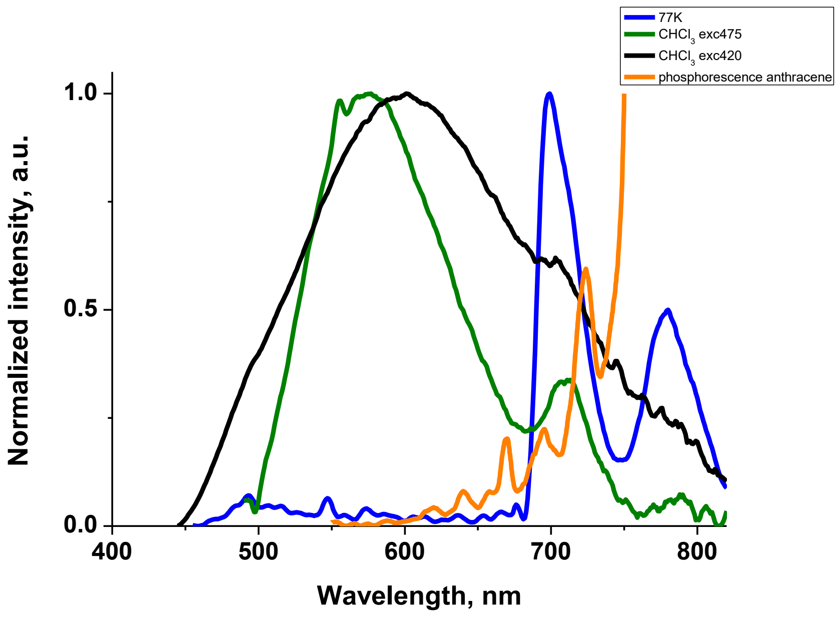
77K
CHCl3 exc475
CHCl3 exc420
phosphorescence anthracene
Normalized intensity, a.u.
Wavelength, nm
0.0
0.5
1.0
400
500
600
700
800

4

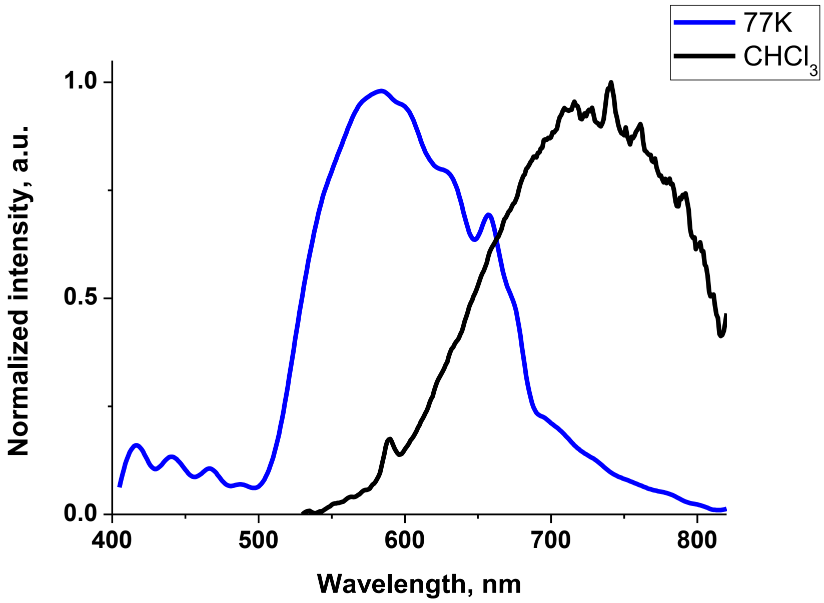
77K
CHCl3
Normalized intensity, a.u.
Wavelength, nm
0.0
0.5
1.0
400
500
600
700
800

5

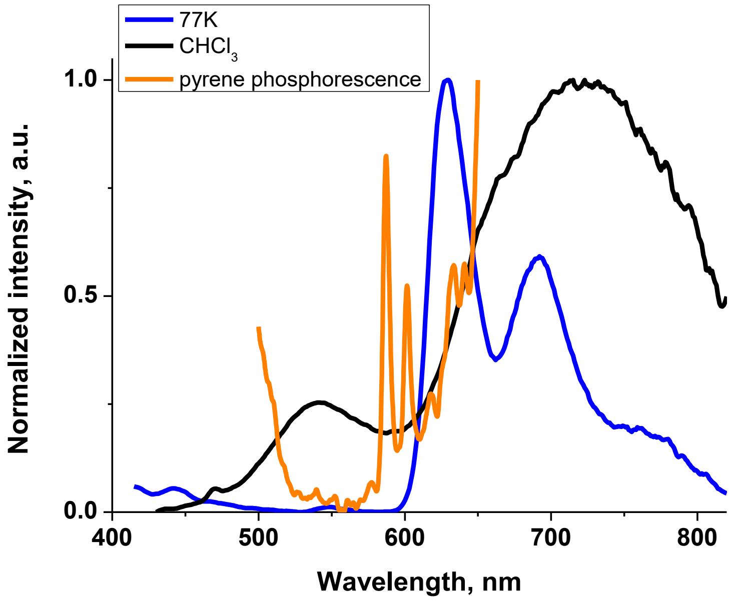
77K
CHCl3
pyrene phosphorescence
1.0
0.5
0.0
Normalized intensity, a.u.
400
500
600
700
800
Wavelength, nm

6

Figure S19. Steady-state spectra (top) and PL decay curves with fits (bottom) of **1** in deaerated $CHCl_3$ solution at room temperature

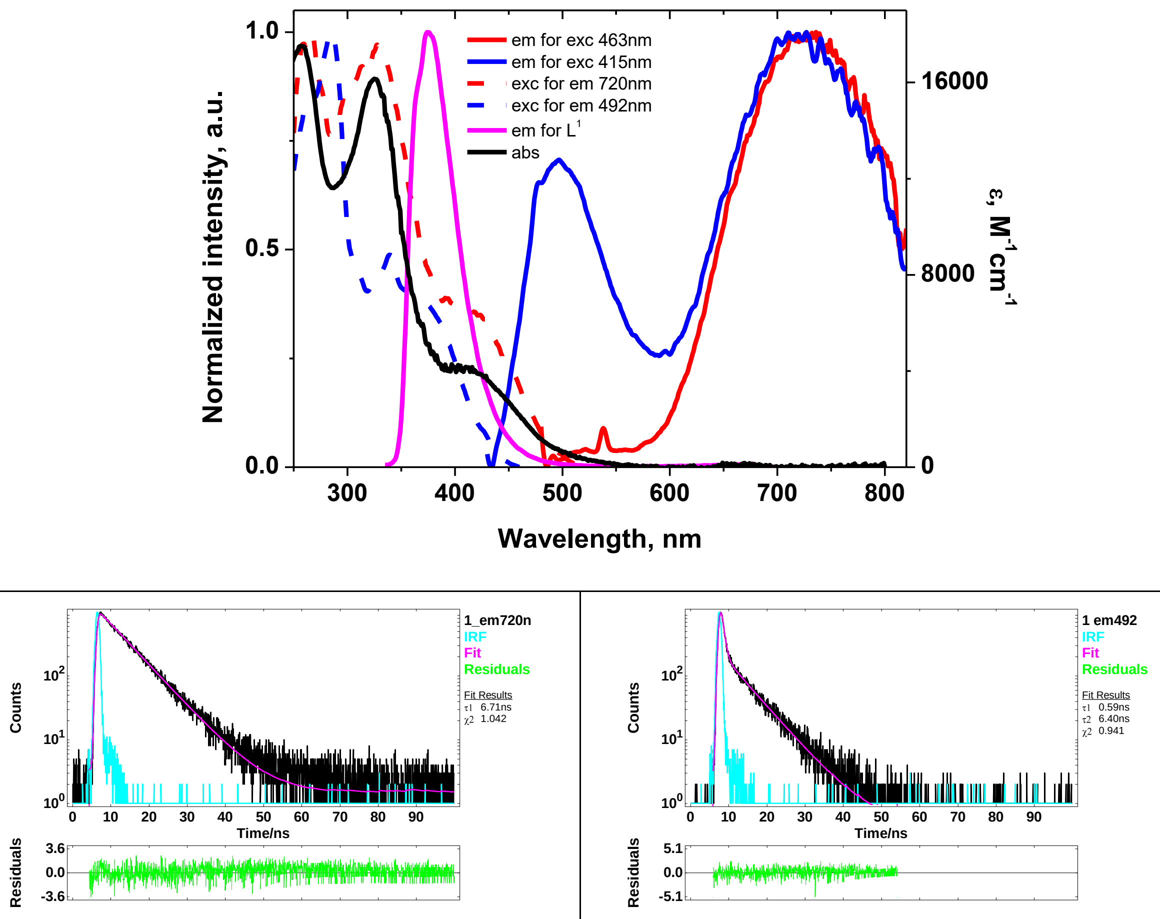

τ	6.71 ns	τ	0.59 ns	6.40 ns
% I	100	% I	47.42	52.58

Figure S20. Steady-state spectra (top) and PL decay curves with fits (bottom) of **2** in deaerated $CHCl_3$ solution at room temperature

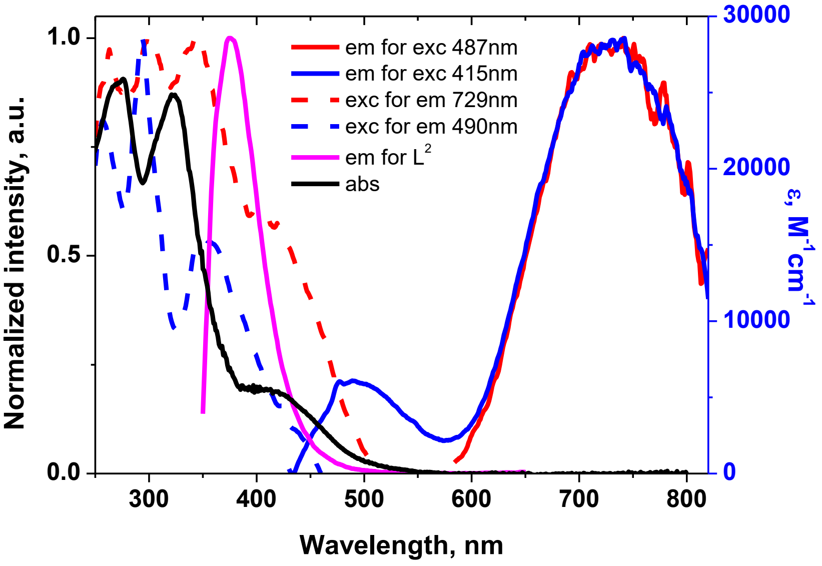

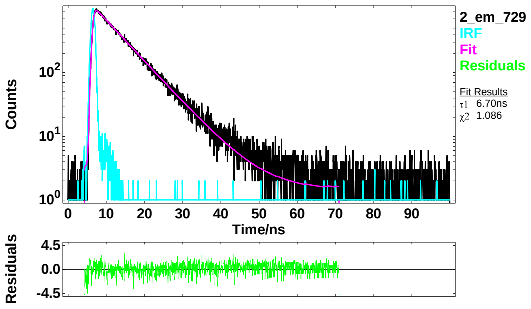

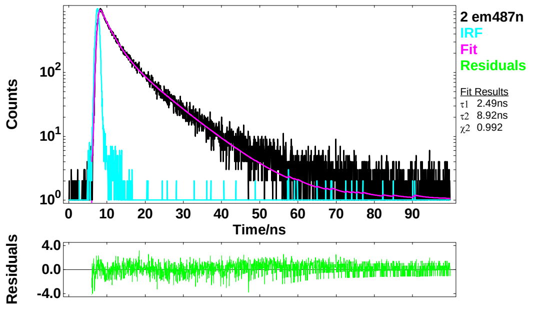

τ	6.70 ns	τ	2.49 ns	8.92 ns
% I	100%	% I	46.75	53.25

Figure S21. Steady-state spectra (top) and PL decay curves with fits (bottom) of **3** in deaerated $CHCl_3$ solution at room temperature

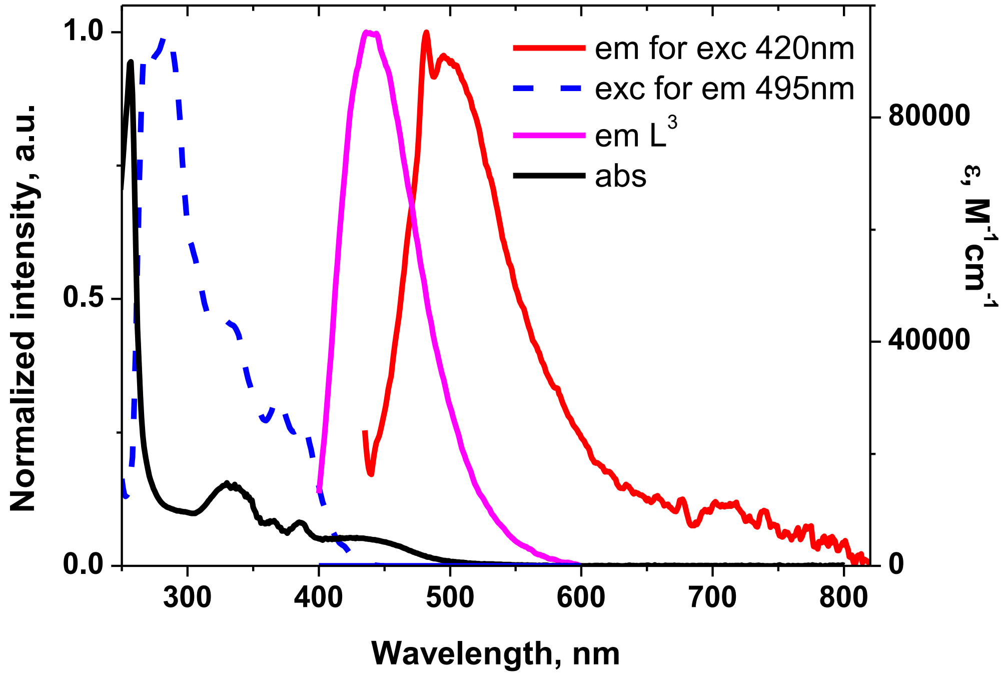

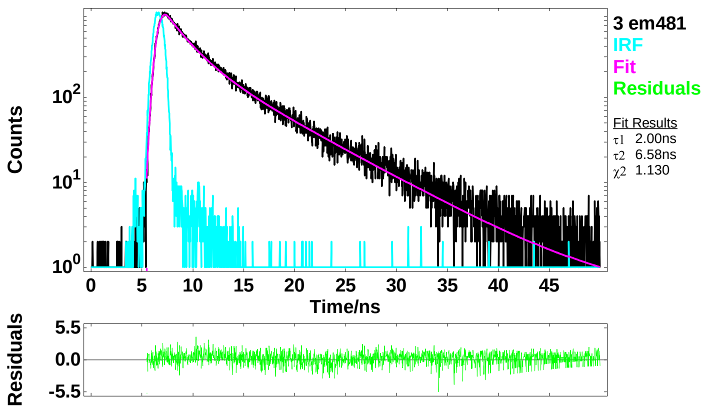

τ	2.00 ns	6.58 ns
% I	42.90	57.10

Figure S22. Steady-state spectra (top) and PL decay curves with fits (bottom) of **4** in deaerated $CHCl_3$ solution at room temperature

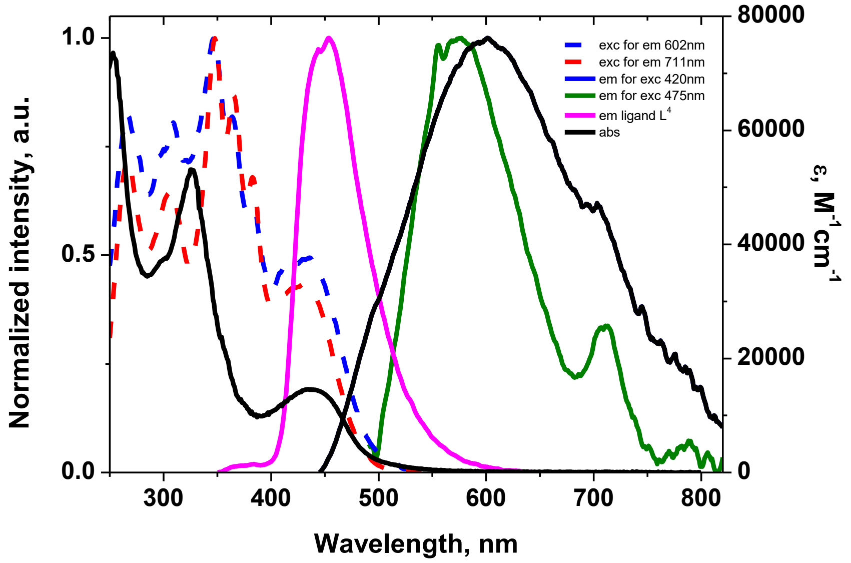

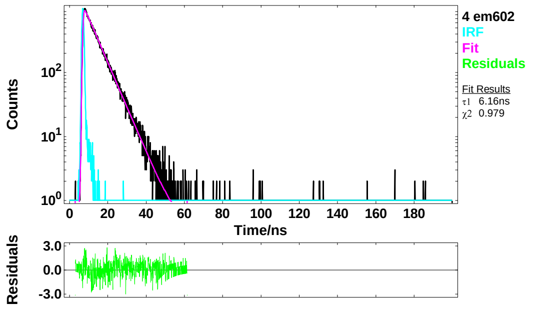

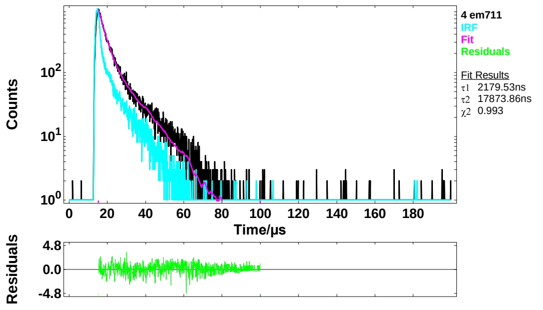

τ	6.16 ns	τ	2179.53 ns	17873.86 ns
% I	100	% I	93.72	6.28

Figure S23. Steady state spectra (top) and PL decay curves with fits (bottom) of **5** in deaerated $CHCl_3$ solution at room temperature

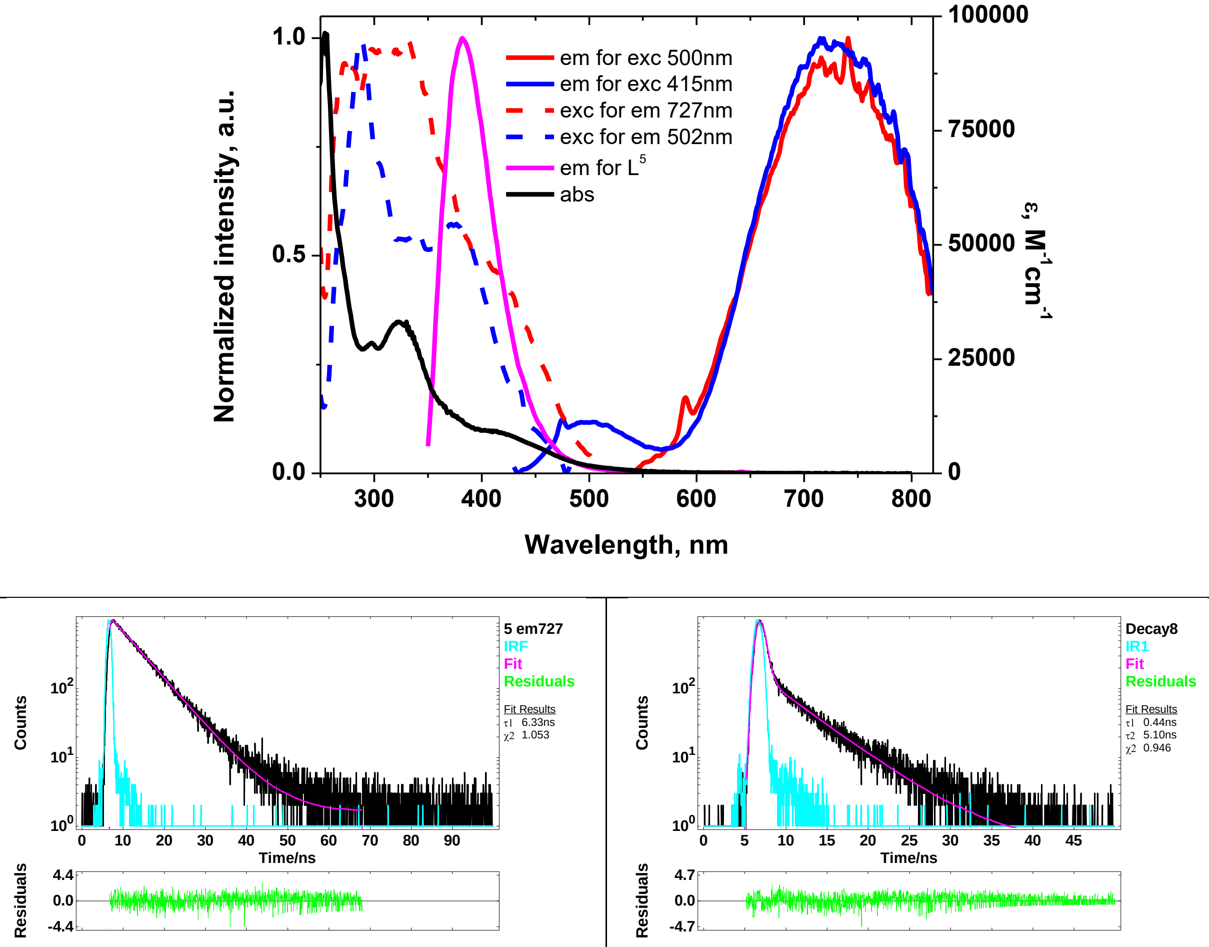

τ	6.33 ns	τ	0.44 ns	5.10 ns
% I	100	% I	62.11	37.89

Figure S24. Steady-state spectra (top) and PL decay curves with fits (bottom) of **6** in deaerated $CHCl_3$ solution at room temperature

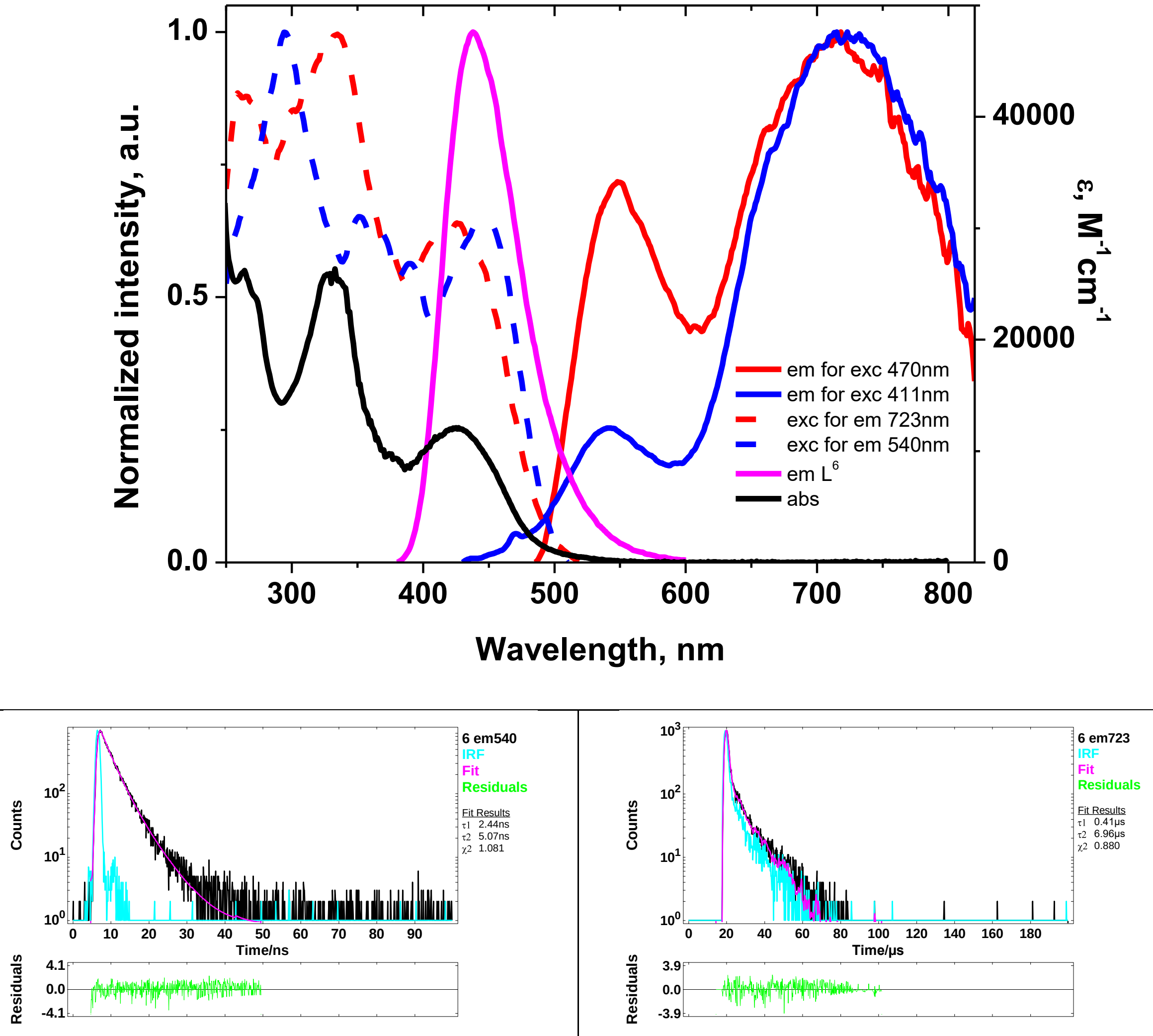

τ	2.44	5.07	τ	0.41 μs	6.96 μs
% I	62.47	37.53	% I	87.89	12.11

Table S16. The PL data for **1**–**6**

**Medium**	**$CHCl_3$**				**77K**			**solid (powder)**				**film**	
**Comp**	**λ_{PE}, [nm]**	**λ_{PL}, [nm]**	**τ_{av}**	**Φ_{PL} (%)**	**λ_{PE}, [nm]**	**λ_{PL}, [nm]**	**τ_{av}**	**λ_{PE}, [nm]**	**λ_{PL}, [nm]**	**τ_{av}**	**Φ_{PL} (%)**	**λ_{PE}, [nm]**	**λ_{PL}, [nm]**
**1**	463	728	6.7 ns	4.25	386	576	16.8 µs	470	667	59.8	8.84	400	603
	415	492 (I), 719 (II)	3.6 ns (I)										
**2**	487	731	6.7 ns	3.72	386	533 sh, **573**	10.3 µs	465	626	97.9	3.55	400	621
	415	490 (I), 729 (II)	5.9 ns (I)										
**3**	475	-	-	6.04	403	499, 538, **599**, 650 (sh) (I)	4.8 µs (I)	No emission				No emission	
	420	495	4.66 ns			**691**, 766 (II)	5045.8 µs (II)						
**4**	475	575 (I), 711 (II)	3.16 µs (II)	5.73	446	**700**, 778	1996.8 µs	No emission				No emission	
	420	602	6.16 ns										
**5**	500	728	6.3 ns	0.82	385	586	4.5 µs	No emission				No emission	
	415	502 (I), 727 (II)	2.2 ns (I)										
**6**	411	540 (I) 723 (II)	3.43 ns (I) 1.20 µs (II)	6.11	413	**629**, 692, 761	2626.0 µs	No emission				452	675

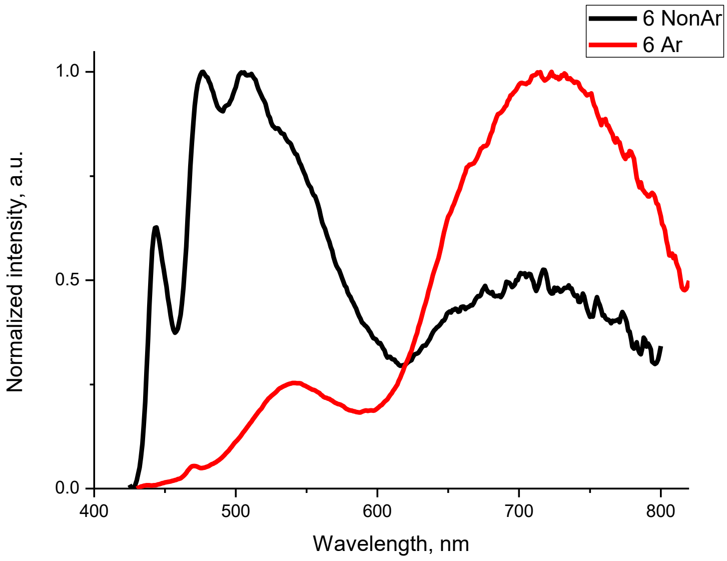

Figure S25. Normalized steady-state emission spectra of **6** in aerated (black line) and deaerated (red line) $CHCl_3$ solution

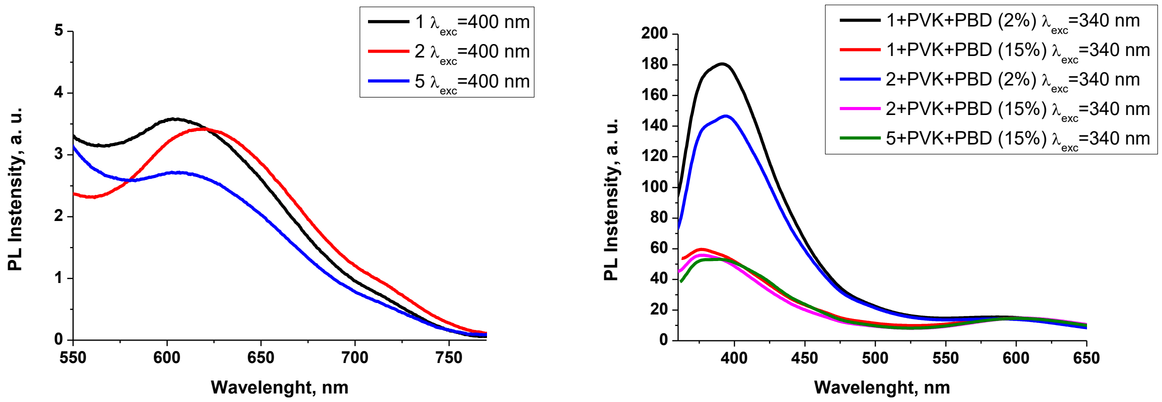

Figure S26. PL spectra of complexes **1**, **2** and **5** as thin films and in PBK:PBD matrix

fsTA

Figure S27. Fluence dependence (top) and photodamage tests (bottom) for **1**, **3**–**4**, **6**

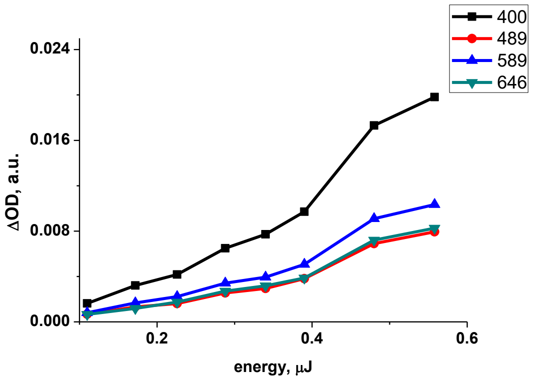 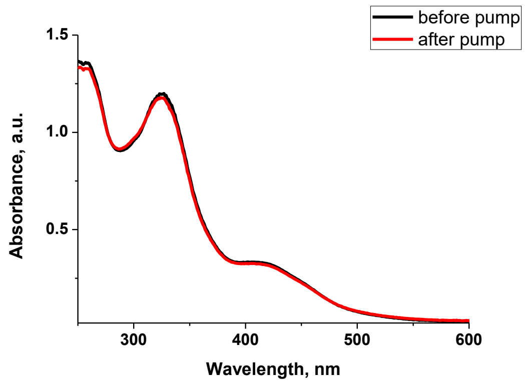	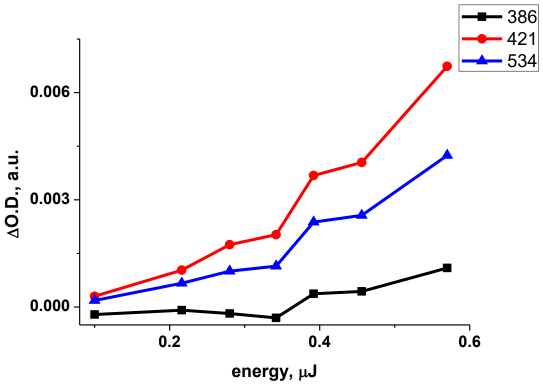 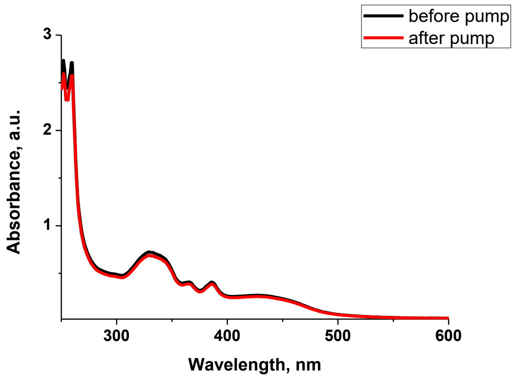
**1 (0.17 μJ per pulse)**	**3 (0.22 μJ per pulse)**

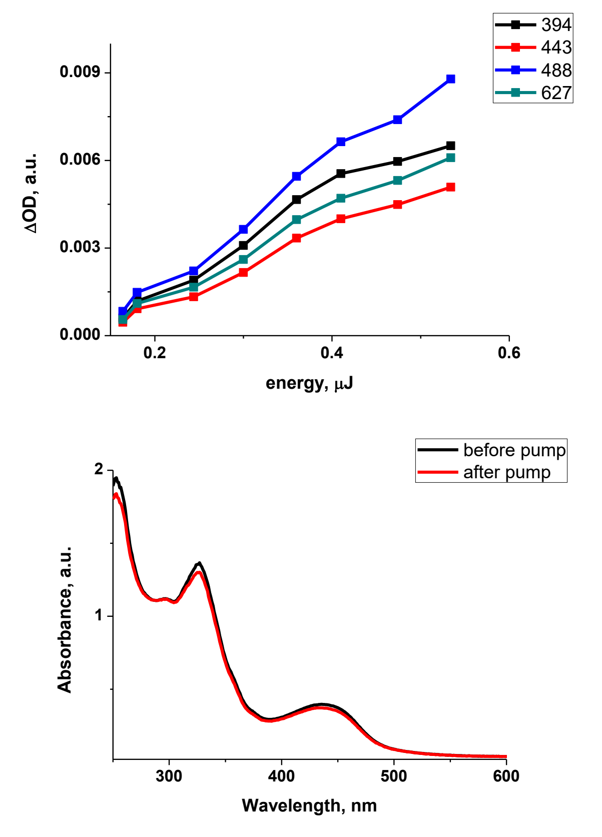

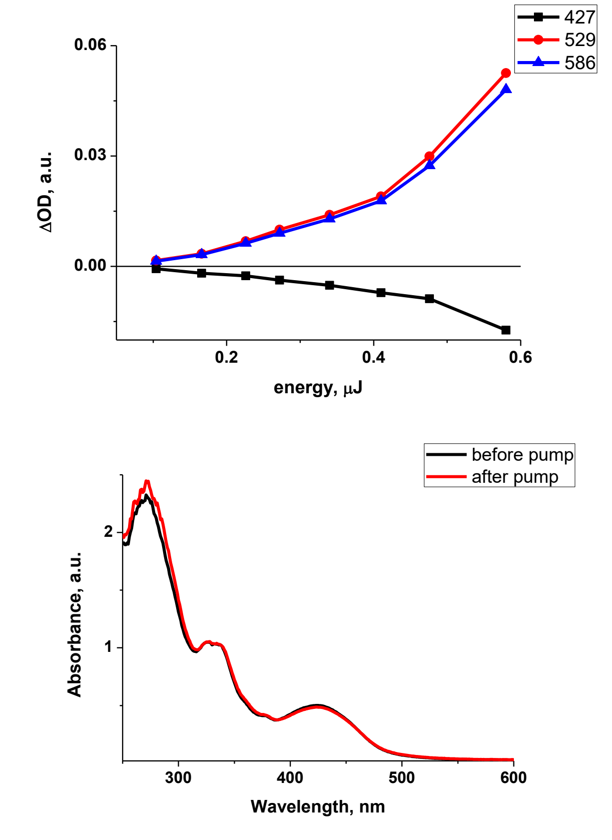

**4 (0.26 μJ per pulse)**	**6 (0.20 μJ per pulse)**

In parenthesis the values of energy per pulse chosen for fsTA experiments are added.

Table S17. Femtosecond transient absorption spectra of **1**, **3**–**4**, **6**

	2D map	Spectra	DAS/EAS	Residuals
**1 in CHCl$_3$**	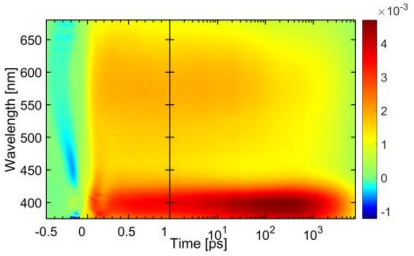	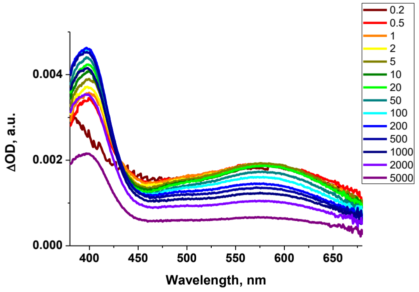	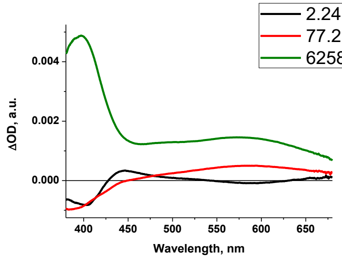 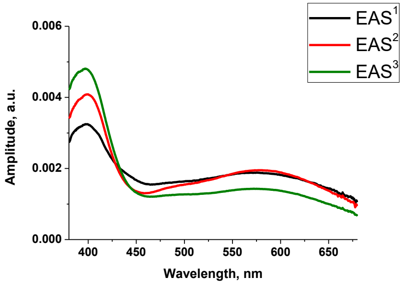	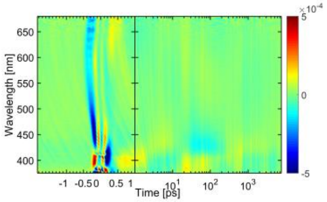
**1 in GlyAc**	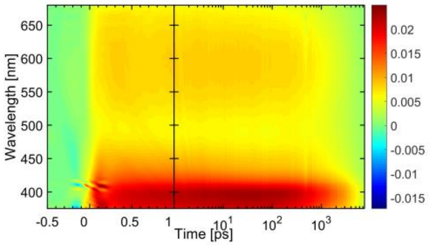	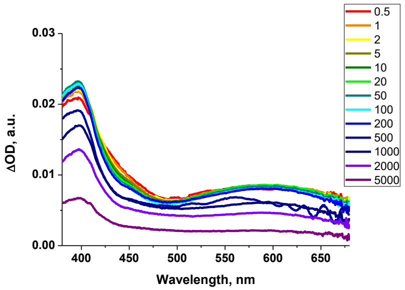	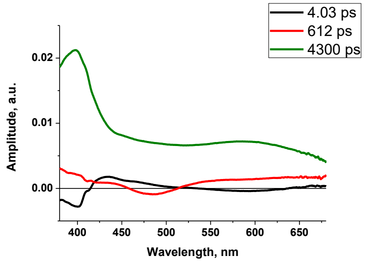	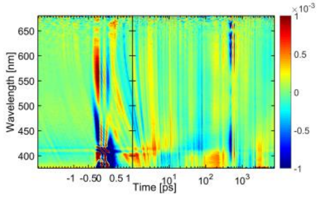

			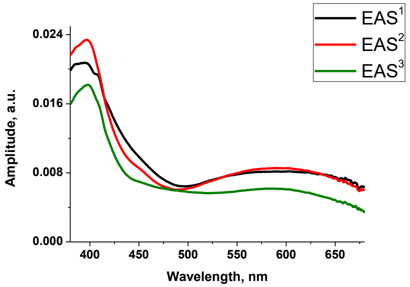 EAS1 EAS2 EAS3 Amplitude, a.u. 0.024 0.016 0.008 0.000 400 450 500 550 600 650 Wavelength, nm	
**3**	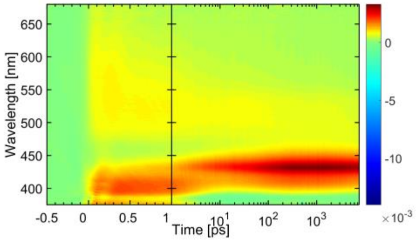 650 600 550 500 450 400 Wavelength [nm] -0.5 0 0.5 1 10^1 10^2 10^3 Time [ps] 0 -5 -10 ×10^-3	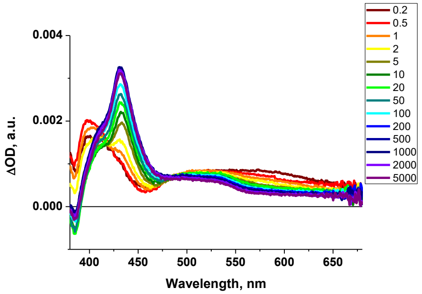 0.2 0.5 1 2 5 10 20 50 100 200 500 1000 2000 5000 ΔOD, a.u. 0.004 0.002 0.000 400 450 500 550 600 650 Wavelength, nm	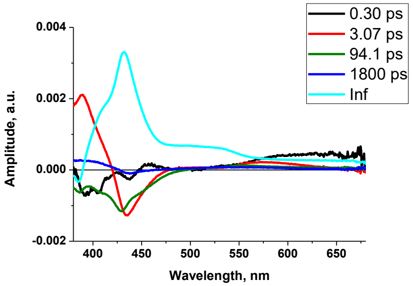 0.30 ps 3.07 ps 94.1 ps 1800 ps Inf Amplitude, a.u. 0.004 0.002 0.000 -0.002 400 450 500 550 600 650 Wavelength, nm 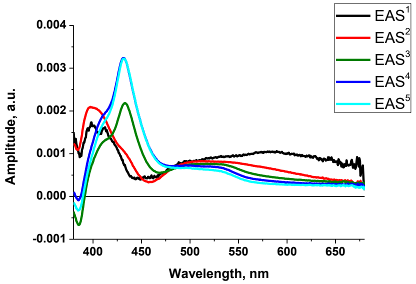 EAS1 EAS2 EAS3 EAS4 EAS5 Amplitude, a.u. 0.004 0.003 0.002 0.001 0.000 -0.001 400 450 500 550 600 650 Wavelength, nm	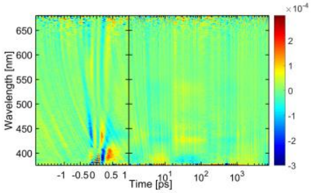 Wavelength [nm] Time [ps]

**4**	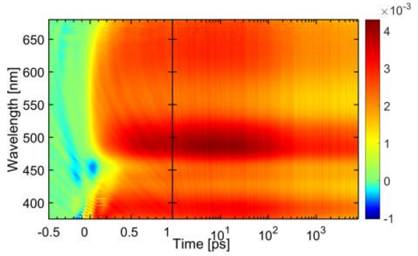Wavelength [nm]<br>Time [ps]<br>×10⁻³	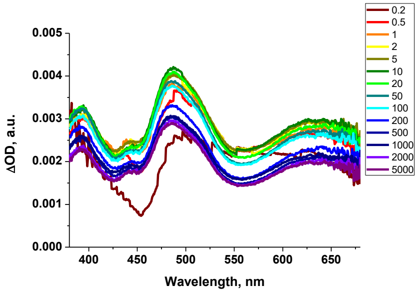ΔOD, a.u.<br>Wavelength, nm<br>0.2<br>0.5<br>1<br>2<br>5<br>10<br>20<br>50<br>100<br>200<br>500<br>1000<br>2000<br>5000	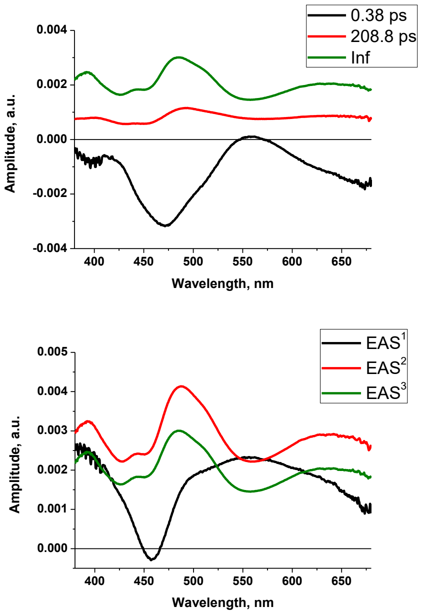0.38 ps<br>208.8 ps<br>Inf<br>Amplitude, a.u.<br>Wavelength, nm<br>EAS1<br>EAS2<br>EAS3<br>Amplitude, a.u.<br>Wavelength, nm	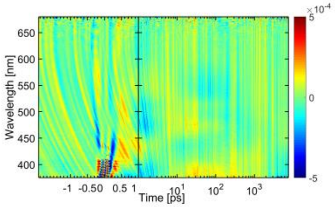Wavelength [nm]<br>Time [ps]<br>×10⁻⁴

6

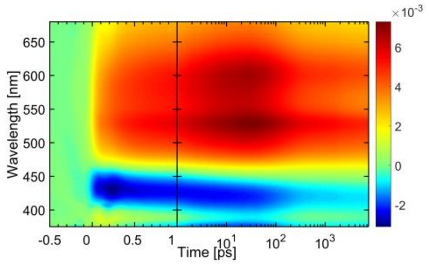
Wavelength [nm]
650
600
550
500
450
400
-0.5
0
0.5
1
Time [ps]
×10-3
6
4
2
0
-2

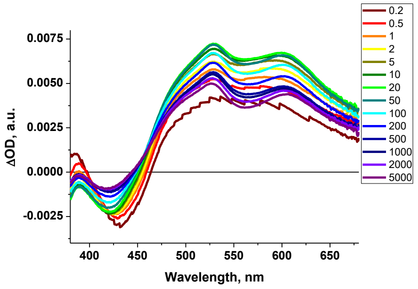
ΔOD, a.u.
0.0075
0.0050
0.0025
0.0000
-0.0025
400
450
500
550
600
650
Wavelength, nm
0.2
0.5
1
2
5
10
20
50
100
200
500
1000
2000
5000

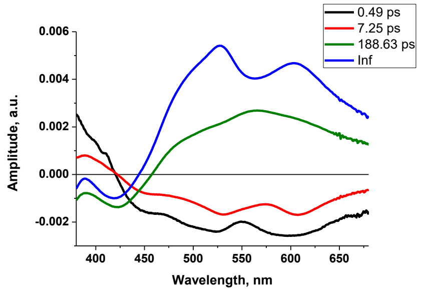
0.49 ps
7.25 ps
188.63 ps
Inf
Amplitude, a.u.
0.006
0.004
0.002
0.000
-0.002
400
450
500
550
600
650
Wavelength, nm

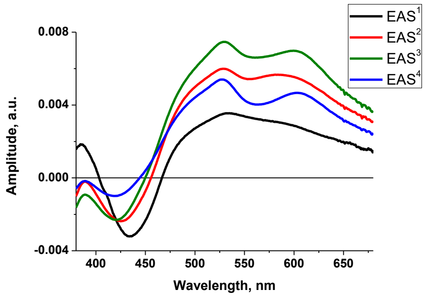
EAS1
EAS2
EAS3
EAS4
Amplitude, a.u.
0.008
0.004
0.000
-0.004
400
450
500
550
600
650
Wavelength, nm

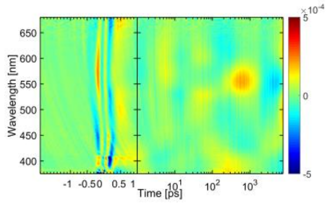
Wavelength [nm]
650
600
550
500
450
400
Time [ps]
5
0
-5

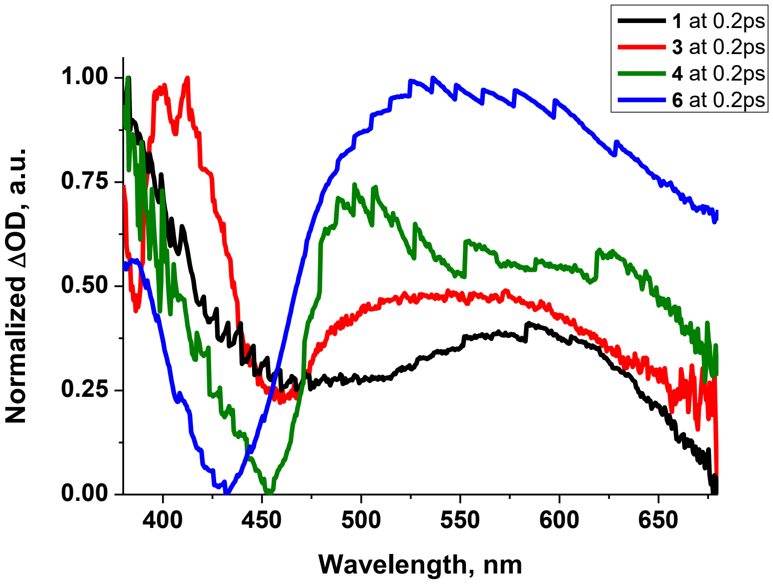

Figure S28. Comparison of normalised fsTA spectra at 0.2ps of **1, 3–4** and **6**
